# Bioactivity study and metabolic profiling of *Colletotrichum alatae* LCS1, an endophyte of club moss *Lycopodium clavatum* L.

**DOI:** 10.1371/journal.pone.0267302

**Published:** 2022-04-28

**Authors:** Hiran Kanti Santra, Debdulal Banerjee

**Affiliations:** Microbiology and Microbial Biotechnology Laboratory, Department of Botany and Forestry, Vidyasagar University, Midnapore, West Bengal, India; Universidad Autonoma de Chihuahua, MEXICO

## Abstract

Endophytes are silent microbial partners of green plants that ensure hosts’ survival in odd conditions. They are known as the factories of multipotent metabolites with diverse bioactivities beneficial to modern pharmaceuticals industry. Endophytic fungi have been screened from a variety of plants and it is the first-time endophytes of club moss is being studied for production of antibacterial and antioxidative compounds. The present study reveals that *Lycopodium clavatum* L. harbors a potent niche of bioactive endophytic fungi and *Colletotrichum alatae* LCS1 was the prime producer of antibacterial and antioxidative compounds among them. The minimum inhibitory and bactericidal concentrations of ethyl- acetate culture extract ranged from 15.62 to 250 μg/mL against four Gram negative and three Gram positive microorganisms including methicillin resistant *Staphylococcus aureus* (ATCC-33591). Bio-autogram based screening followed by Gas chromatographic analysis confirmed the occurrence of 17 bioactive compounds and α-bisabolol is known to be the prime one. Alfa bisabolol is a unique and versatile bioactive essential oil and facilitates variety of functions. Killing kinetics data along with leakage of macromolecules into extracellular environment supports the cidal activity of the antibacterial principles at MBC values. Isolate *C*. *alatae* LCS1 was optimized by one variable at a time system coupled with response surface methodology for broad spectrum antibacterial production. The organism yielded maximum response (22.66±0.894 mm of zone of inhibition against MRSA) in 250 mL Erlenmeyer flask containing 50 mL potato dextrose broth supplemented with (g/L) glucose, 7.53; yeast extract concentration, 0.47; NaCl, 0.10 with medium pH 6.46; after 134 hours of incubation at 26°C. Optimized fermentation parameters enhanced antibacterial activity up-to more than 50% than the pre-optimized one (10.33±0.57 mm). Endophytic LCS1 was also efficient in free radical scavenging tested by DPPH, ABTS, H_2_O_2_ and FRAP assay with an IC_50_ values of 23.38±5.32 to 82.873±6.479 μg/mL.

## Introduction

In 21st century AMR (antimicrobial resistance) of MDR (multidrug resistance) strains has been a fatal issue to be treated with strict and efficient hands. Multidrug resistance of pathogenic microorganisms is rising day by day questioning the safety and security of human lives from dreadful diseases. AMR is roughly estimated to cause 10 million deaths annually and dropping of GDP (gross domestic production) up to US$ 100 trillion by the end of 2050 dragging almost 24 million people to below poverty line [1]. Antimicrobials like antibiotics, antivirals, antifungals become inefficacious towards bacterial, fungal and viral pathogens or parasites due to antibiotic selection pressure, natural or anthropogenic induced mutation caused by irrational, extreme use of this chemotherapeutic agents, community or nosocomial infections etc. The well-established 1st, 2nd and 3rd generation antibiotics are losing their superior activity gradually, MIC values against dreadful pathogens are deteriorating day by day, common diseases like respiratory tract infections, sexually transmitted diseases, urinary tract infections are taking huge tolls, strains are becoming un-susceptible and soon will gain resistance and the situation is becoming a global concern. So, it is high time to switch to some new and novel options and endophytic fungi or actinobacteria derived secondary bioactive metabolites from efficient but unpopular age old ayurvedic medicinal plants are a very wise option in this regard. Another severe problem of today’s world is oxidative stress as a result of altered life styles. Oxidative or nitrosative stress is generated due to disturbance in the homeostasis of oxidant-antioxidant molecules as a result of rapid increase of ROS (reactive oxygen species) and RNS (reactive nitrogen species). Higher levels of oxidative stress increase disease susceptibility, and accelerates the disease onset and progression. The exogenous and endogenous antioxidative molecules restore the balance by scavenging free radicals and also by upregulating antioxidative defences. Antioxidative molecules are miraculous in nature reducing stress issues, solving aging related problems and also minimizing the chances of malignancy or proliferation of cancer cells etc. in eukaryotic system [2]. Consumption of antioxidants has been a part and parcel of daily diet offering a soulful life and optimized body function. The search of antioxidant is now a days focused on endophytic microorganisms as they are efficient synthesizers of these lifesaving molecules [3–6]. Endophytes of *L*. *clavatum* follow the same trend and are proved to be a remarkable agent for in-vitro antioxidative principles production.

Endophytes are known to be the plant symbionts which access nutrients, shelter from plant, and in return protects plant from biotic or abiotic stress situations [7,8]. These diverse community (fungi, bacteria or actinobacteria) synthesize unique metabolomes (alkaloids, aliphatic compounds, polyketides, phenylpropanoids, quinones, flavonoids, peptides, phenols, steroids, hydrocarbon derivatives- biofuels or myco-diesel components. etc.) with multifaceted biopotentials e.g.- antimicrobials, antioxidants, anti-cancers etc. which validates them as widely acceptable in the field of agriculture, pharmaceuticals industry and in leading biotechnological sectors [9–13].

Majorly angiospermic and gymnospermic plants are explored widely for endophyte related study and cryptograms are a bit neglected in this respect [14–16]. We recently reported the drought stress ameliorating action of β-glucan rich heteropolysaccharide from the endophytic *Colletotrichum alatae* LCS1 isolated from *Lycopodium clavatum* [17].

In the present investigation antibacterial activity of the endophyte *C*. *alatae* LCS1 (from *L*. *clavatum* collected from Tapobon forest of West Bengal, India) is evaluated against seven pathogenic strains (including MRSA) and antibacterial components are optimized to scale up production. Here in total 17 metabolites with antimicrobial and antioxidative property are reported. NIST library-based identification has revealed bisabolol as the prime bioactive metabolites along with long chain alcohols, phenols and organic acids. Bisabolol is an all- square component with antibacterial, antioxidative, anti-irritant, leshmenicidal activity etc. [18]. This study for the first time reports the bisabolol production from any *Lycopodium clavatum* derived endophytic source.

## Materials and methods

### Study site and collection of plant specimen

Disease free, healthy and mature plants (*Lycopodium clavatum* L.) with fruit bodies were collected in zipper-lock polythene from undisturbed forest patches of Tapobon of Paschim Medinipur district, West Bengal, India (Latitude 22°25/ to 22°57/N, longitude 87°11/E, Altitude 23 M) having tropical warm and humid climate with mean temperature of 34°C and average rainfall of 120 cm. Samples were brought to the Microbiology and Microbial Biotechnology Laboratory, Vidyasagar University for further study of endophytic fungal flora.

### Isolation of the endophytic fungi

The plant was surface sterilized first in running tap water and then in sodium hypochlorite-NaOCl (2–10%), H2O2-hydrogen peroxide (3%) solution for the removal of surface microorganisms [19]. Plant parts like strobilus, stem and leaf were selected as explants for endophyte isolation. They were cut into small pieces by sterile blades and were flamed in spirit burner for surface drying after emerging in 70% alcohol for 5–10 s. Strobilus was separated into two equal halves and angular cuts of different portions or tissues were made. Explants were placed on water agar and incubated in BOD incubator at 30° C for 3 to 5 days. The effectiveness of this sterilization and isolation process was cross checked by the explant imprintation technique described by Schulz et al. [19]. After 48 to 72 h of incubation the endophytic fungal hyphae emerge from explant tissues. Agar blocks with fungal hyphae was transferred to potato dextrose agar medium for further identification studies.

### Identification of isolated endophytes

After 3–5 days of incubation period, endophytic fungi were identified according to their plate and microscopic morphology. Micro-photographs of the sexual and asexual spores were taken after staining them with lactophenol, cotton blue and mounted in cover slip using light microscope (Nikon Eclipse LV100 POL and Leica DM 3000) as a tool for identification. Standard literatures were used for identification purpose [20]. The isolate devoid of any sexual or asexual bodies was also grown on CLA (carnation leaf pieces agar) medium in order to induce reproductive structure formation and was identified as *Colletotrichum alatae* LCS1 (GenBank acc. no.-MH102383.1) by Santra and Banerjee [17].

### Scanning electron microscopy (SEM)

For the better understanding of the fungal hyphal structures scanning electron microscopy was done after proper pre-treatment and appropriate desiccation of the fungal sample. The samples were slowly dehydrated in different gradations of ethanol ranging from 10% to 90% to avoid the shrinking of the mycelial structures and then critically point dried, coated with gold and examined with a Zeiss EVO18 scanning electron microscope following the standard protocols [21].

### Preparation of plant extracts

Fresh leaves, stems and strobilus of *L*. *clavatum* were collected from Tapobon region (Paschim Medinipur, West Bengal, India) were brought to laboratory in an ice box, washed in tap water followed by rinsing with distilled water. The plant parts were sundried for 24 to 72 h. 2 gm of whole plant extract (in powder form) was dissolved in 100 mL water, stirred at 150 rpm for 30 min at 25°C and filtered through Whatman filter paper (No. 4). The aqueous form of plant extract (2%w/v) with a final concentration of 20 mg/mL was mixed with nutrient agar medium for antibacterial test [22].

### Screening of fungal endophytes for antimicrobial activity

Liquid culture preparation of the endophytes includes the inoculation of the liquid extracts or broth (PDB) in Erlenmeyer flasks with fungal PDA blocks of 1 cm^2^ length and breadth respectively having fungal hyphae. Cell free extracts of the endophytic isolates were first screened by agar well diffusion method for their antimicrobial potential against Gram positive; *B*. *cereus* (ATCC 14579), *B*. *subtilis* (ATCC 11774), Methicillin resistant *Staphylococcus aureus* (ATCC 33591) and Gram negative; *P*. *mirabilis* (ATCC 12453), *P*. *aeruginosa* (ATCC 9027), *V*. *parahaemolyticus* (ATCC 17802), *E*. coli (MTCC 4296) pathogenic bacteria [22]. 50 μL of the cell free culture extract was added to the 5 mm diameter agar well in the nutrient plates previously inoculated with pathogenic bacterial strains and incubated for 24 h at optimum temperature of 28 to 37°C. Streptomycin, clindamycin, vancomycin, ciprofloxacin was used as standard antibiotic. Clear zones of inhibition was measured and the potent isolates were subjected to further study. The screening for antimicrobial production was performed in two steps. The first step includes the determination of the antibacterial activity of the endophytic culture extract only and the second step includes the antibacterial potential determination of the culture extract along with the whole plant extract. Obtained results were compared in order to investigate the interactive effect of endophytic fungi and plant compounds in their antibacterial property [22].

### Determination of MIC and MBC values by micro-dilution method

MIC values for four solvents (ethyl acetate, ethyl ether, methanol and methanol: water (1:1, v/v)) were tested for both mycelial extract and culture broth (cell free extract) to evaluate the occurrence of antibacterial compounds whether bound in mycelial cells or expelled outside at culture extract [22]. Erlenmeyer flasks (250 mL) with 100 mL of PDB were inoculated by fungal hyphae for submerged culture and kept in BOD shaker incubator for 4 to 5 days in dark conditions and in an agitation of 120 rpm. Filter-paper based filtration was done after incubation followed by the centrifugation for the separation of the fungal mycelium from broth. The culture broth was mixed with two to three times of ethyl acetate, methanol, methanol: water, ethyl ether and agitated for 40–60 min. The two layers of liquid were separated using separating funnel and the extraction was repeatedly done for two to three times. Removal of solvent is mediated by Bacca Buchi Rota vapor at 70 rpm and 46°C with 230 vacuum pressures. The extracts were further dissolved in methanol and subjected to determination of MIC and MBC values. MIC values of the several extracts were determined by a broth micro-dilution assay in PDB medium at 25°C. Different concentrations (1.9 μg/mL-2 mg/mL) of ethyl-acetate extract were prepared and added to fresh nutrient broths containing 1% of bacterial culture (when OD620nm = 0.5) for the determination of MIC and MBC values. The MBC values for each pathogenic bacterium were used to elucidate the bactericidal property of the antibacterial principles by marking the drastic change of CFU between the control and the treated one over time. The change in CFU values were recorded in a graph for clear understanding as a time killing kinetics [23]. The recorded MIC and MBC values were the lowest concentrations of the crude extracts that have bacteriostatic and bactericidal activity respectively. The results were expressed in unit of μg/mL and presented as the mean of three independent experiments.

#### Effect of LCS1 extract on bacterial cell integrity

To detect the mode of action of the active principles of the isolate LCS1 the quantification of leaked extracellular macromolecules in bacterial culture were done. Freshly harvested active bacterial cultures were washed and re-suspended in Na-P buffers followed by the treatment of MIC for 6 to 24 h. Each set was centrifuged and spectro-photometrical quantification was done for detection of DNA and protein content in a comparison with the control set [24,25].

### Optimization for the production of antibacterial compound

#### Optimization by OVAT method

To determine the best cultural conditions regarding the antibacterial production, the valuable parameters like incubation temperature, fermentation time, medium composition in terms of different types and concentration of carbon and nitrogen sources, medium pH was needed to be optimized [26]. For proper determination of these values the endophytic fungi were grown in Erlenmeyer flaks for different incubation periods (1–10 days), with different incubation temperature (22–30°C) and then with different medium pH (4.0–7.0). To elucidate the necessity of additional nutrients for increase of biomass and antibacterial production, variety of carbon (1 g/L, w/v starch, fructose, glucose, maltose) and nitrogen (0.3 g/L w/v tryptone, yeast extract and NH4NO3) sources were used additionally in the PDB medium. The appropriate concentrations (2–12% for sugar and 0.1 to 0.9% for nitrogen source) of the two obtained best sugar and nitrogen sources were determined corresponding to their maximum biomass and antibacterial production. Different types of ionic salts (0.05 g/L, w/v NaCl, KCl, MgCl2, CaCl2) were also used to evaluate their influence on antibacterial production. The effect of oxygen availability on antibacterial production was determined by analyzing the surface area, medium volume, head space volume, total volume and also medium depth in Erlenmeyer flask [27].

#### Optimization using Box-Behnken design

Using the OVAT (One variable at a time) data further construction was made using RSM (Response surface methodology). The investigational design adopted was a Box–Behnken experimental design with four factors taken from one variable at a time outcomes. The design deals with four independent factors each at three different levels of −1.0 (lower than the optimum), 0 (at the optimum value) and +1.0 (higher than the optimum one). Antibacterial production is subjected to a second order polynomial equation by using a multiple regression technique. A factual model concerned to the most significant factors was also obtained. The system performance was defined by the subsequent second order polynomial equation: Y = β0 208 + ΣβiXi + ΣβijXiXj +ΣβiiX^2^ where Y was known as the predicted response or dependent variable, xi and xj were here independent factors, β0 is represented as the intercept of the regression equation, βi was called as the linear coefficient, βii was indicated as the quadratic coefficient and βij was designated as the interaction coefficient [28].

#### Biomass estimation

Endophytic culture broth was centrifuged at 10,000 × g for 10 min and mycelial biomass was collected, dried at 55°C for 24 h. The dry weight of mycelial biomass was measured.

### Post optimization estimation of bisabolol

The relative amount of bisabolol produced in the culture extract was estimated after comparing clear zone of inhibitions produced by different concentrations of the standard (−)-6-Methyl-2-(4-methyl-3- cyclohexen-1-yl)-5-hepten-2-ol (chemical name of bisabolol-(Supelco) Sigma Aldrich) at different phases of optimization. Concentration of bisabolol or also called as levomenol in culture filtrates was determined as amount of bisabolol (volume of culture filtrate) loaded into wells of MHA (Mueller-Hinton Agar) plates. The amount of bisabolol produced (μg/mL) is further confirmed by HPLC based standard curve calibration [29]. Volume of well was calculated from the equation: V = π r^2^h, where r = radius and h = height of well (π) (0.25)^2^ 0.5 [well diameter = 0.5 cm and height = 0.5 cm]

### Purification and characterization of the active compounds

#### TLC and bioautography

Thin layer chromatography was done by using varying ratios of polar and non-polar (Methanol- chloroform) solvents as the mobile phase. Plate with clear band of separation was poured with agar mixed bacterial strain (MRSA) for bioautography technique [30]. Antibacterial activity was detected by applying the agar-overlaid plate with TTC (2%) solution.

#### HPLC chromatogram of the purified compound

After optimization, bisabolol was subjected to HPLC (Agilent Technologies 1200 series) analysis using a C18 reverse phase column and gradient elution was used with the mobile phase composed of (A) acetonitrile–water–phosphoric acid (19:80:1) and (B) acetonitrile. The flow rate of the eluent was 0.8 mL/min with an UV detection at 200 nm [29]. The amount of bisabolol produced in pre- and post-optimized condition is calculated from the standard calibration curve of the bisabolol. Different concentrations of bisabolol are run on HPLC system and the respective area count is noted to develop the calibration curve.

#### GC-MS of the active fraction

Semi purified crude extract was dissolved in 3 mL of methanol (GC grade, Hi-media) and analyzed by single quadrupole GC (TRACE 1300)-MS (ISQ QD Single Quadrupole) system-Thermo scientific with ESI mode. The instrument was configured with a DB-5 Ultra Inert column (30 m length and 0.25 mm inner diameter) for 22 min run of 1 μL sample (split-less flow) with an injector port and oven temperature of 240°C and 50°C respectively having 10°C/min ramping time up to 260°C with helium as the carrier gas. The flow velocity of the carrier gas was set at 1 mL/minute. The ionization source was kept at 250°C with 70 eV of ionization energy and 0.1 kV ionization current. Mass fragmentation pattern was analyzed by X-Calibur software. The identification of the various compounds was based on the SI and RSI value with the best matched compound in the NIST library [9].

### Determination of antioxidative potential of the endophytic culture extract

#### DPPH radical scavenging assay

The free radical scavengability of fungal ethyl acetate extracts were measured by using DPPH (diphenyl picryl hydrazyl) [31]. 200 μL of 0.1 mM of DPPH solution (prepared by dissolving DPPH in methanol) was added to different concentrations of EA extracts (0, 10, 25, 50, 100 μg mL^−1^) of fungal isolates. The two liquids were mixed thoroughly and incubated for thirty minutes at dark conditions at normal room temperature. Post incubation tasks include the measurement of absorbance at 517 nm of wavelength using UV spectrophotometer. EA (ethyl acetate) mixed with methanolic DPPH was used as blank. The radical scavenging potential was calculated in the form of percentage using the formula = [1-(Abs S1/ Abs S2)] * 100. Abs S1 = absorbance of the sample at 517 nm; Abs S2 = absorbance of the control at 517 nm. Straight line equation was plotted and IC50 value was calculated from that equation. Different concentration (6.25–100 μg mL^−1^) of ascorbic acid was used as a positive control and IC50 value were determined.

#### ABTS radical scavenging assay

The ABTS (2, 2’-azino-bis 3-ethylbenzothiazoline-6-sulfonic acid) was prepared by mixing aqueous ABTS (2 mM) with potassium persulfate (2.45 mM) and stored in room temperature for 24 hours at dark. Prior to detection of antioxidative activity of the different concentrations (12.5 μg/mL-100 μg/mL) of culture extracts or standard, the ABTS solution was set at an absorbance of 0.750 ± 0.025 at 734 nm diluting with sodium phosphate buffer (0.1 M, pH 7.4). Mixing of diluted ABTS with culture extract was followed by 30 minutes incubation and the absorbance was measured at 734 nm. The scavenging capability of ABTS radical was calculated by the following equation: (1- (A1/ A2) * 100) where, A1 was the initial concentration of the ABTS and A2 was absorbance of the remaining concentration of ABTS in the presence of culture extract or standard [32]. Straight line equation was plotted and IC50 value was calculated from that equation. Different concentration (6.25–100 μg mL^−1^) of ascorbic acid was used as a positive control and IC50 value was determined.

#### H2O2 scavenging activity

Different concentrations of endophytic culture extracts (20–100 μg/mL) were tested for their H2O2 scavenging activity following the methods of [33]. Various concentrations of the culture extracts mixed in phosphate buffer (0.1 M, pH 7.4) were added to 43 mM H2O2 and the absorbance was measured at 230 nm. Blank one was devoid of culture extract. H2O2 scavenging percentage was obtained by the following equation (1- A1/ A2) * 100 here, A1 and A2 were represented as the absorbance of the control and different concentrations of culture extract or standards (ascorbic acid) [34]. Straight line equation was plotted and IC50 value was calculated from that equation. Different concentration (6.25–100 μg mL^−1^) of ascorbic acid was used as a positive control and IC50 value was determined.

#### Fe3+ (Ferric ions) reducing antioxidant power assay (FRAP)

Endophytic culture extracts (25–400 μg/mL) were evaluated for their reducing power following the methods of [35]. Ethyl-acetate culture extracts were mixed with sodium phosphate buffer (0.2 M, at pH 6.6), potassium ferricyanide [K3Fe (CN)6] (1%) and incubated at 50°C for 20 min. Post incubation procedure includes addition of 10% trichloroacetic acid, doubled distilled water, 0.1% FeCl3 and absorbance was measured at 700nm. Higher OD values indicate enhanced reducing power and ascorbic acid was used as a standard. Straight line equation was plotted and IC50 value was calculated from that equation. Different concentration (6.25–100 μg mL^−1^) of ascorbic acid was used as a positive control and IC50 value was determined.

### Statistical analysis

All experiments were performed in triplicate and the results were presented as means± standard errors (SE). Data were analyzed by Prism GraphPad software version 9.2.0 (332) (San Diego, California, USA). Minitab (version 20.2) statistical software was used for Response surface methodology experiments (Box Behnken Design).

## Results

### Identification of endophytic isolates of *Lycopodium clavatum*

**Fig 1:**
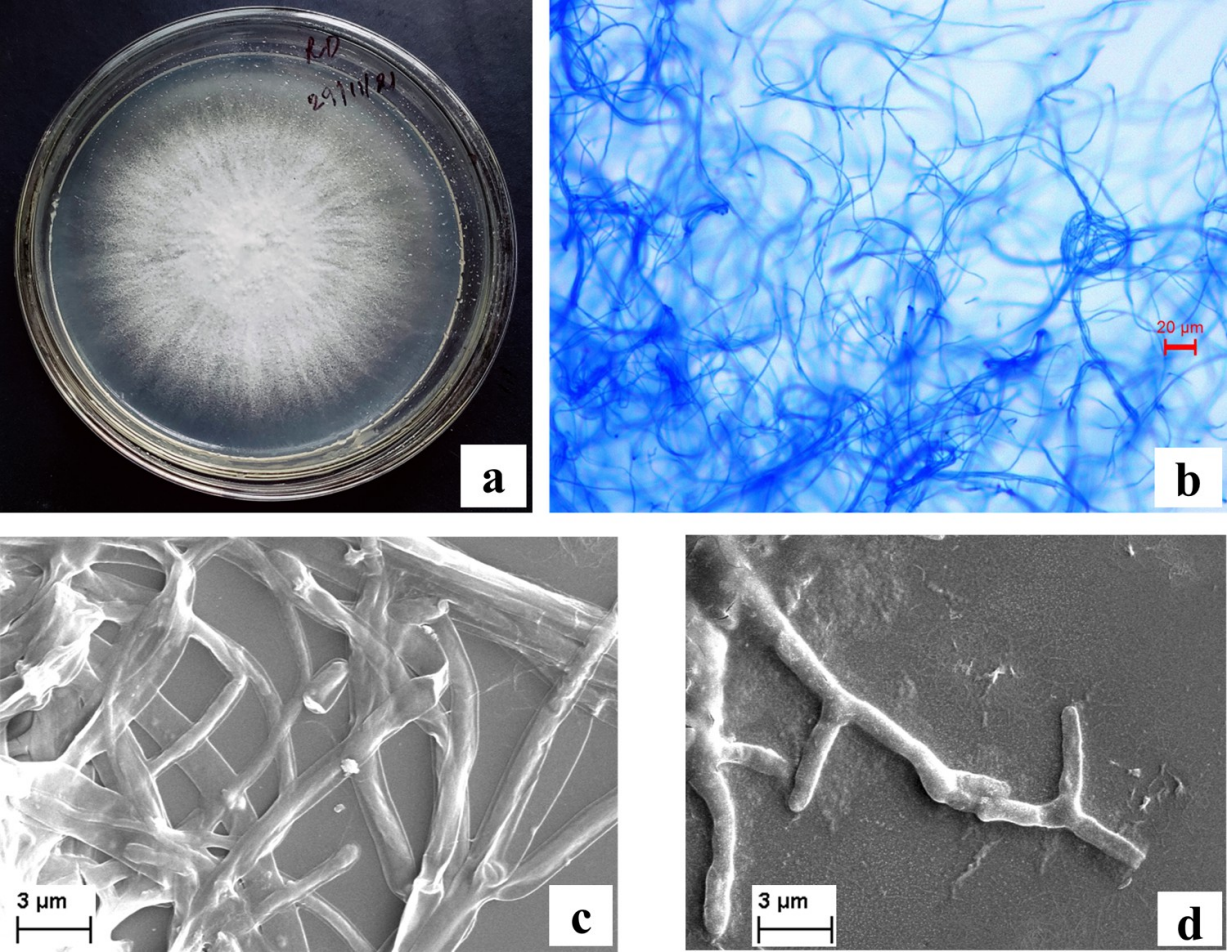
10 days old culture of *Colletotrichum alatae* LCS1 (A), Light microscopic image of sterile mycelium (B), Scanning electron micrograph of sterile hyphae (C-D).

In total six endophytic fungi were isolated from different tissues of *Lycopodium clavatum*. Out of the six only one isolate did not produce any reproductive structure and other five showed prominent sexual or asexual bodies. The plate morphology with top and bottom view was taken along with their light and scanning electronic microscopic observations (Fig 1). The sterile one that has only vegetative mycelia was previously identified as *Colletotrichum alatae* LCS1 (GenBank acc. no.-MH102383.1) by rRNA analysis (Santra and Banerjee 2022). Other five isolates were identified as *Scopulariopsis* sp. *Lasidiplodia* sp. *Pestalotiopsis* sp., *Phomopsis* sp. *Phoma* sp. (S1 Fig).

### Antimicrobial activity of endophytes

#### Primary screening for antibacterial activity

**Fig 2:**
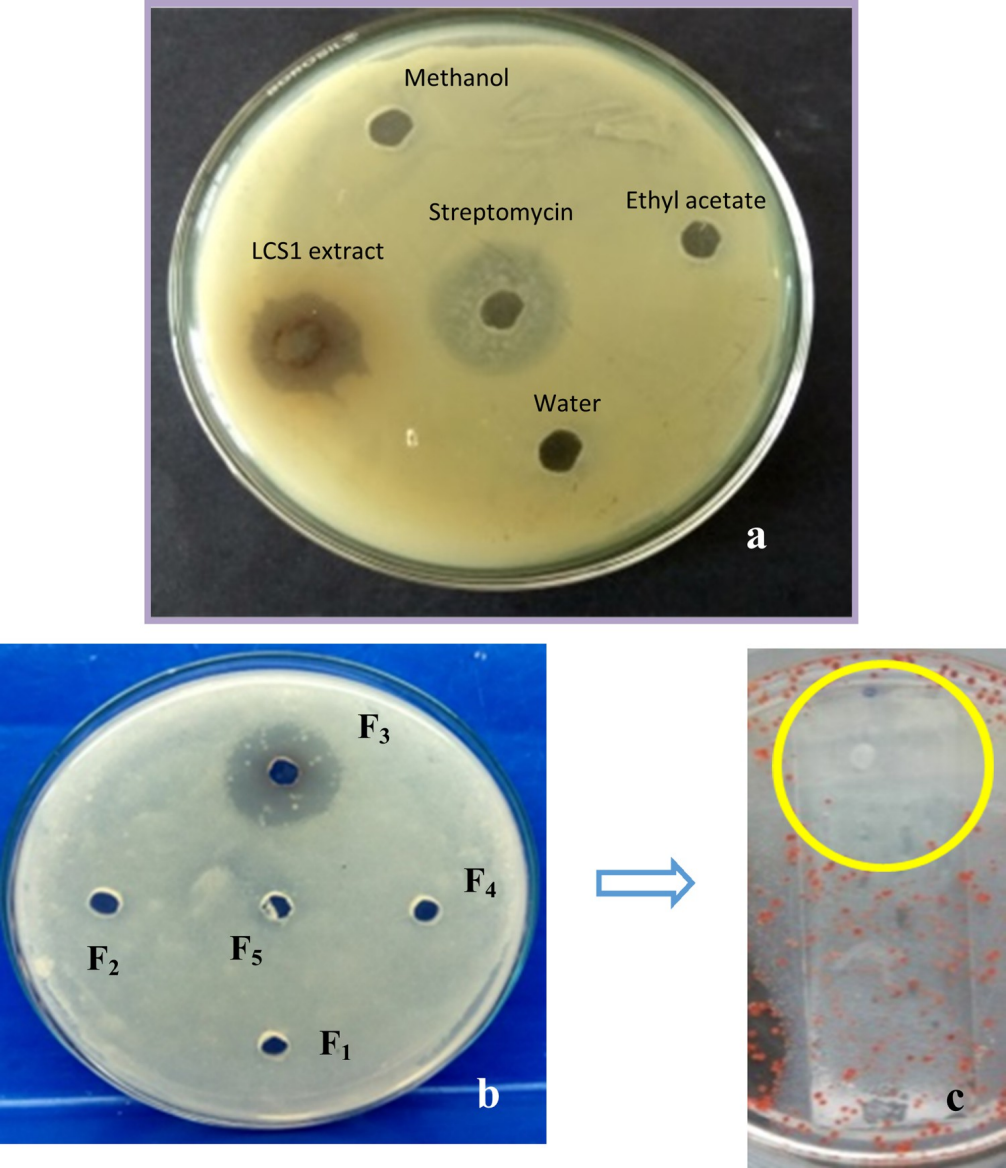
a- Antibacterial activity of the LCS1 culture extract (31.25 μg/mL) against MRSA in a nutrient agar medium. b- Antibacterial action of the F3 (fraction 3) with highest clear zone of inhibition. c- Clear zone of inhibition of bacterial (MRSA) growth on TLC plate after TTC application.

Antibacterial activity of the endophytic isolates was determined by agar well diffusion techniques against three pathogenic strains of Gram-positive bacteria and four Gram negative bacteria. Antibacterial activity of the isolates in their water extract were measured and recorded in S1A Table. All the six isolated endophytic fungi (*Colletotrichum alatae* LCS1., *Phoma* sp., *Phomopsis* sp., *Pestalotiopsis* sp., *Lasidiplodia* sp., *Scopulariopsis* sp.) showed broad spectrum antibacterial activity. Finally, the best three (*Colletotrichum alatae* LCS1., *Phomopsis* sp., *Lasidiplodia* sp.) antibacterial producers, confirmed in terms of formation of clear zone of inhibition, were studied further. *Colletotrichum alatae* LCS1 was the most efficient producer of antibacterial compounds and exhibited the maximum clear zone of inhibition (Fig 2A). Enhancement of antibacterial activity of culture extracts had been seen when the media was supplemented with plant extract along with nutrient agar (S1A Table). Antibacterial activity of the endophytic isolate *Colletotrichum alatae* LCS1 got doubled when the growth media is supplemented with plant extract in case of *Bacillus cereus*. In case of other two Gram positive bacteria (*B*. *subtilis* and MRSA) and one Gram negative bacteria (*P*. *mirabilis*) the cumulative effect showed an enhancement of antibacterial activity up-to 8.5, 6.9 and 5.5 times respectively. Ethyl acetate fractions of *Phomopsis* sp. culture extract yielded 100%, 90%, and 65% better antibacterial activity against *P*. *mirabilis*, *B*. *subtilis*, MRSA respectively when punched up with plant extract. Another isolate *Lasidiplodia* sp. also showed a synergistic effect on antibacterial activity when coupled with plant extract and the clear zone of inhibition of bacterial growth got enhanced up-to 100%, 72% and 66% against *P*. *aeruginosa*, *B*. *cereus* and MRSA respectively.

#### Selection of extraction agent and determination of MIC values

The three endophytic isolates were mass cultured and bioactive components were extracted using four different (ethyl acetate, ethyl ether, methanol, methanol: water) types of solvents for the evaluation of antibacterial potentiality. Out of the three isolate LCS1 (*Colletotrichum alatae* LCS1) was proved to be the most efficient producers of antibacterial compound and MIC values for endophytic *Colletotrichum* sp. strain LCS1 in different solvents were recorded (S1B Table). Out of the four solvents ethyl-acetate was found to be the most effective solvent in terms of extracting bioactive components from the culture broth and the MIC values with standard antibiotics are presented in Table 1.

**Table 1:** Antibacterial activity (MIC and MBC- μg/mL) of *C*. *alatae* LCS1 EA (ethyl-acetate) culture extract against pathogenic bacteria.

Pathogenic bacteria	MIC (μg/mL)					MBC (μg/mL)				
	LCS1 extract	CPFX	STREP	CLINDA	VANCO	LCS1 extract	CPFX	STREP	CLINDA	VANCO
*B*. *cereus* (ATCC 14579)	15.62 (a, a1)	7.81 (b)	15.62 (a)	3.90 (c)	0.97 (d)	31.25 (a, a2)	15.62 (b)	31.25 (a)	7.81 (c)	1.95 (d)
*B*. *subtilis* (ATCC 11774)	15.62 (a, a1)	7.81 (b)	15.62 (a)	3.90 (c)	0.97 (d)	31.25 (a, a2)	15.62 (b)	31.25 (a)	7.81 (c)	1.95 (d)
MRSA (ATCC 33591)	31.25 (a, a1)	15.62 (b)	62.5 (c)	7.81 (d)	3.90 (e)	62.5 (a, a2)	31.25 (b)	125 (c)	15.62 (d)	7.81 (e)
*P*. *mirabilis* (ATCC 12453)	31.25 (a, a1)	15.62 (b)	62.5 (c)	7.81 (d)	3.90 (e)	62.5 (a, a2)	31.25 (b)	125 (c)	15.62 (d)	7.81 (e)
*P*. *aeruginosa* (ATCC 9027)	62.5 (a, a1)	3.90 (b)	7.81 (c)	1.95 (d)	0.48 (e)	125 (a, a2)	7.81 (b)	15.62 (c)	3.90 (d)	0.97 (e)
*V*. *parahaemolyticus (ATCC 17802)*	62.5 (a, a1)	3.90 (b)	7.81 (c)	1.95 (d)	0.48 (e)	125 (a, a2)	7.81 (b)	15.62 (c)	3.90 (d)	0.97 (e)
*E*. *coli (MTCC 4296)*	125 (a, a1)	3.90 (b)	7.81 (c)	1.95 (d)	0.48 (e)	250 (a, a2)	7.81 (b)	15.62 (c)	3.90 (d)	0.97 (e)

The five different letters (a, b, c, d, e) indicates potential statistical differences between the five testing agents- LCS1 and other four antibiotics regarding their MIC and MBC values respectively. The other two letters a1 and a2 indicates valid statistical difference between the MIC and MBC value of LCS1 extract (row wise).

MIC values of the culture extract and the mycelium extract of *Colletotrichum alatae* LCS1 on different solvents ranged from 15.62 μg/mL to 2 mg/mL. Ethyl acetate culture extract is the most effective against all the tested pathogens with the lowest MIC value of 15.62 μg/mL against Gram positive *Bacillus cereus* and *Bacillus subtilis*, moderate value of 31.25 μg/mL against Methicillin resistant *Staphylococcus aureus* (causal agent of skin infections, pneumonia, heart valve infections, and bone infections in human). Gram negative pathogenic strains like *Proteus mirabilis* (causal organism of urinary tract infections in human), *Pseudomonas aeruginosa*, *Vibrio parahaemolyticus* and *Escherichia coli* were inhibited by 31.25 μg/mL, 62.5 μg/mL and 125 μg/mL of *Colletotrichum alatae* LCS1 culture EA extract respectively.

Out of the four solvents tested for the extraction of antibacterial compounds only two; ethyl acetate and ethyl ether extracts were effective widely against all the microorganisms. Other- wise methanolic extracts were only effective against *Bacillus cereus* and *Bacillus subtilis* pathogens with a MIC value of 250 μg/mL and 500 μg/mL respectively for culture broth and mycelial extracts. Methanolic culture extracts have higher abilities to inhibit bacterial growth in comparison to their mycelial extracts against *Bacillus subtilis* and *Bacillus cereus* with a MIC value of 250 μg/mL (for culture extract) and 500 μg/mL (for mycelial extract). Results were dissimilar in case of methanol: water (1:1) based extraction of antibacterial compound having higher MIC values of 500 μg/mL, 1.00 mg/mL for mycelial extracts and 1.00 mg/mL, 2 mg/mL for culture extracts against *B*. *cereus* and *B*. *subtilis* respectively. In the rest of the tests mycelial extracts have shown dissatisfactory MIC values in comparison to culture extracts (S1B Table).

#### Effect of LCS1 extract on bacterial growth kinetics and leakage of macromolecules

Different concentrations of the EA culture extract (1.9 to 2000 μg/mL) were added to the active bacterial cultures (OD625) and the change in OD values were measured after 24 h of treatment along-side with the documentation of the CFU/mL of each treated set. A decrease in CFU/mL number upon increase in EA extract concentration is seen in case of all the tested pathogenic micro-organisms. At certain concentrations CFU numbers got drastically changed to sub-minimal level and zero respectively. The concentrations at which no colonies were observed were marked as Minimum inhibitory concentration values and the concentration at which no visible bacteria are found on sub-plating is known as Minimum bactericidal concentration.

**Fig 3:**
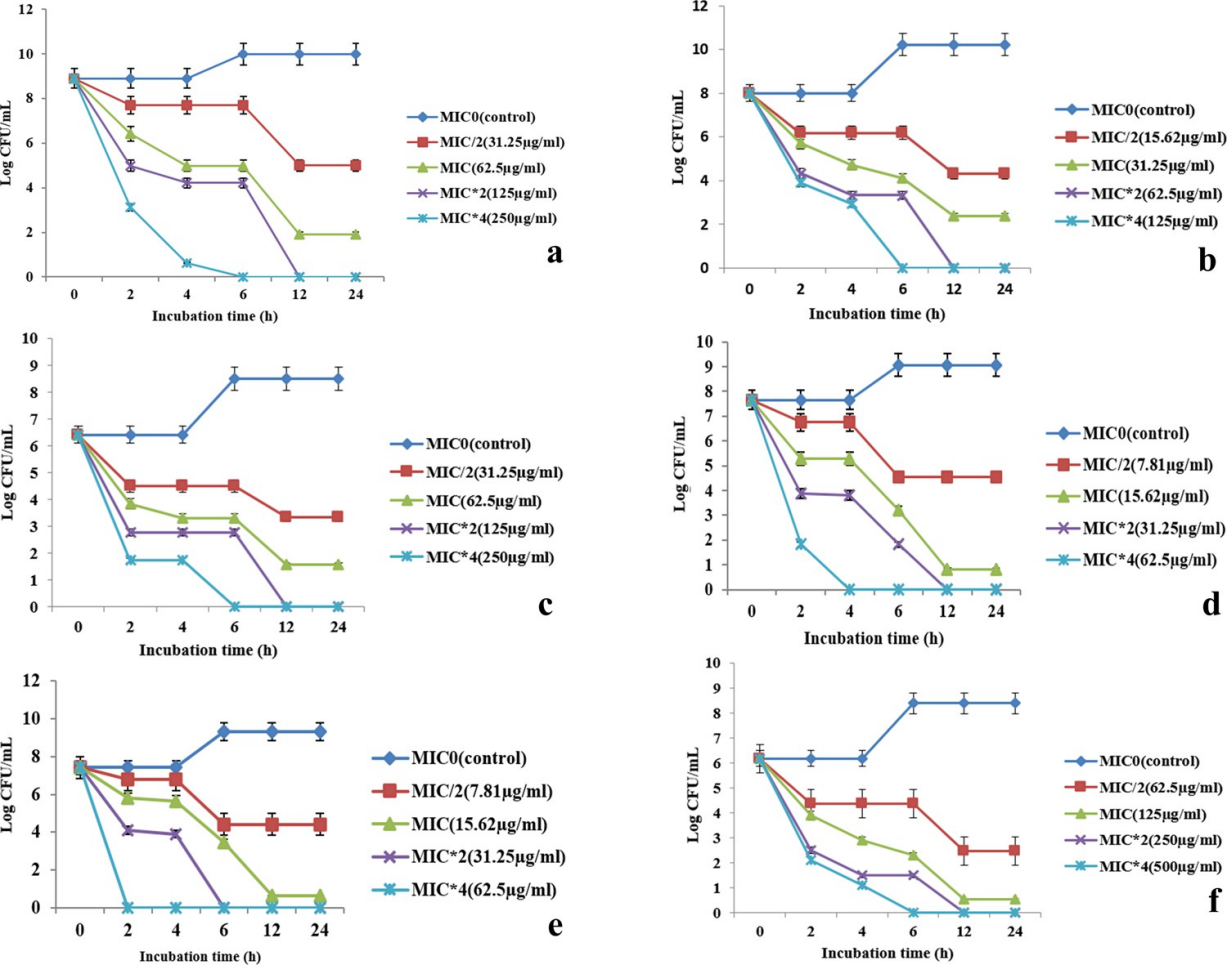
Killing kinetics of pathogenic microorganisms over time when treated with different concentrations of MIC values a–*P*. *aeruginosa* b–MRSA c- *V*. *parahaemolyticus* d- *B*. *cereus* e–*B*. *subtilis* f–*E*. *coli*. Values on the graphs are the means ± Standard error (SE) of the three replicates.

The culture extract (ethyl-acetate fraction) affected the eradication of the tested bacterial pathogens in a concentration dependent manner. The observations of the time-kill kinetics were summarized in detail in Fig 3. Results were indicative of the fact that antibacterial principles produced by the fungal endophyte caused a sharp decrease on viable counts of all the bacterial pathogens to 0 log 10 CFU/mL. Especially the MRSA strain used in this study found to be highly affected by the antibacterial components. MIC and MBC values for *Bacillus subtilis* and *Bacillus cereus* were same (15.62 and 31.25 μg/mL). The MIC and MBC values were also same for MRSA, *Proteus mirabilis* (31.25 and 62.5 μg/mL) and also, for *Vibrio parahaemolyticus* and *Pseudomonas aeruginosa* (62.5 and 125 μg/mL). According to the varying values of MIC and MBC for all the seven pathogenic strains different degree of killing kinetics were observed. It could be concluded that relatively low concentrations of antibacterial components are needed for inhibition of Gram positive one than the Gram-negative pathogens. Compounds were bactericidal in nature as the CFUs reduced to zero values and substantial number of cells were eradicated at low concentrations of 15.62 μg/mL (Against *B*. *cereus* and *B*. *subtilis*).

**Fig 4:**
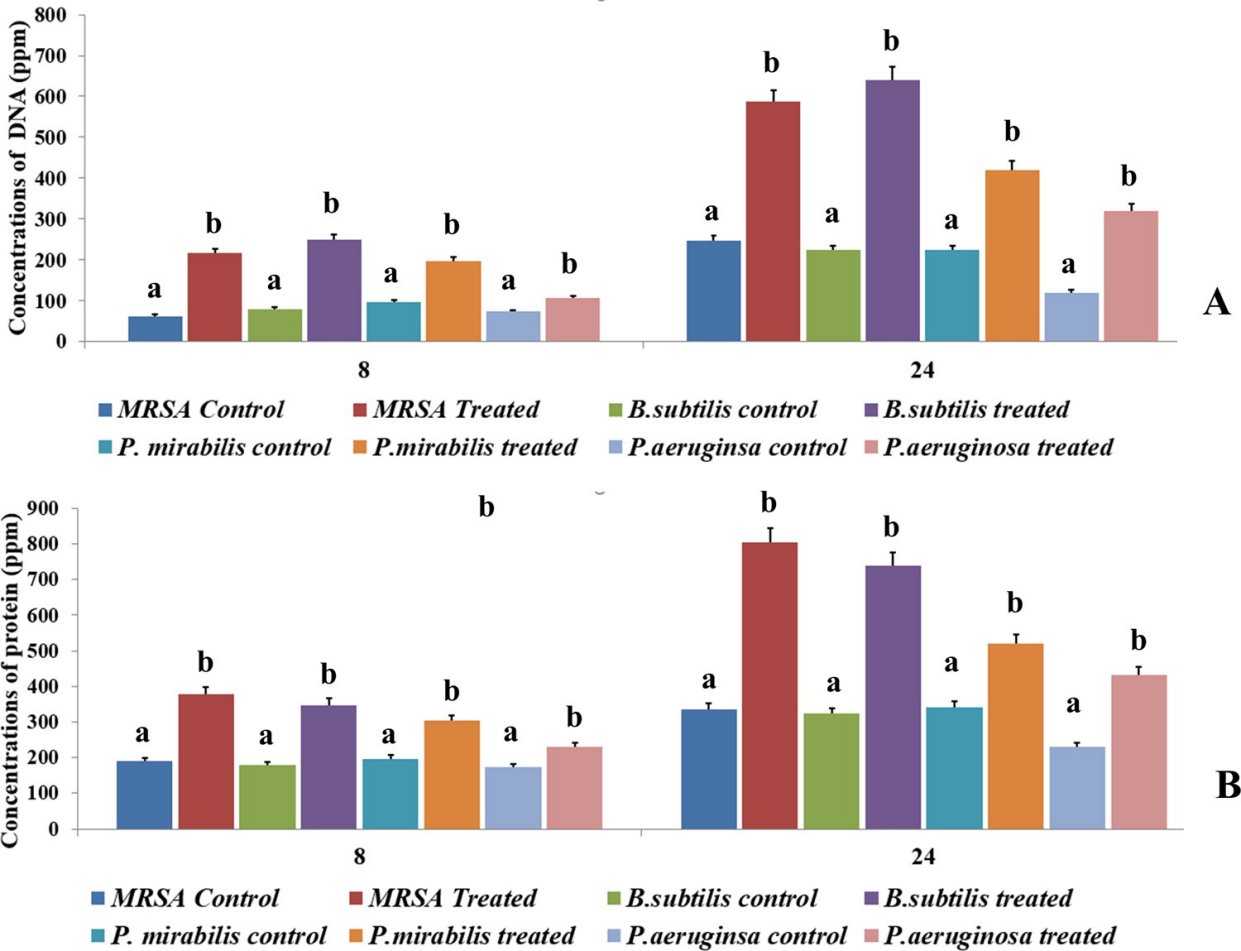
Leakage of intracellular macromolecules in to extracellular environment A–DNA content B- protein content. Values on the graphs are the means ± Standard error (SE) of the three replicates. Tukey’s multiple comparison test was performed. The different letters a, and b in each case (for each bacterial pathogen at control and at treated condition) represents a significant difference between them (At, P<0.05).

The broad-spectrum antimicrobial principles produced by LCS1 leads to leakage of intracellular macromolecules like DNA and protein content that specifies the cidal effect of the active components causing lysis of the treated cells. The cell bursting effect was more prominent in case of Gram-positive pathogens than the Gram-negative ones. Results were satisfying in case of MRSA and *B*. *subtilis* than *P*. *mirabilis* and *P*. *aeruginosa*. There was a twofold increase of DNA and protein content in the extracellular environment leaked out from the treated bacerial cells after 24 h of treatment in a comparison of 10 h treatment (Fig 4).

### Optimization for antibacterial production by OVAT method

#### Optimization of culture conditions

*Colletotrichum alatae* LCS1 was grown on submerged fermentation in 250 mL Erlenmeyer flask for 2 to 10 days. It was found that there was a parallel response of fermentation time on biomass production and antibacterial activity. Up-to, day six both biomass and antibacterial production got enhanced simultaneously but after reaching to the peak at 6th day (biomass 5.343 ± 0.045 g/L and zone of inhibition 9.67 ± 0.58 mm) there was a fall on both the values up to tenth day (Table 2). The probable reason for the sudden fall of both the values could be the entry of fungal isolate into its death phase causing a metabolic block. Antibacterial activity of the isolate is strictly related to incubation temperature. It had been observed that the organism grows well and produces antimicrobial compounds in satisfactory amount at a temperature of 26°C (biomass 7.863 ± 0.030 g/L and zone of inhibition 12.33 ± 0.58 mm). Though *Colletotrichum alatae* LCS1 grows in a temperature range of 22 to 30°C but the optimum activity was reported to be at 26°C and after that the increase in temperature causes a decrease in antibacterial performance as well as biomass production (Table 2). The reason of such negative relationship after a particular range between the biomass estimation and antibacterial production was due to inactivation of some particular enzymes needed for antibacterial production.

**Table 2:** Effect of different physical conditions and chemical supplements on biomass and antibacterial activity (ZOI-zone of inhibition) by *Colletotrichum alatae* LCS1. Against MRSA (Methicillin resistant *Staphylococcus aureus*).

Parameters	Effectors	Percentage added (g/L)	Biomass (g/L)	Antibacterial activity (ZOI in mm)
Incubation time (in day)	2	**-**	3.566±0.057(a)	3.33±0.58(a)
	4	**-**	4.466±0.115(b)	5±0(b)
	6	**-**	5.343±0.045(c)	9.67±0.58(c)
	8	**-**	4.916±0.020(b)	6.33±0.58(d)
	10	**-**	4.133±0.057(d)	6±1(d)
Incubation temperature (°C)	22	**-**	5.970±0.026(a)	8±0(a)
	24	**-**	6.756±0.049(b)	9.33±0.58(b)
	26	**-**	7.863±0.030(c)	12.33±0.58(c)
	28	**-**	4.963±0.046(d)	9.67±0.58(b)
	30	**-**	3.076±0.015(e)	7±0(d)
Initial medium pH	4	**-**	2.286±0.005(a)	2.33±0.58(a)
	5	**-**	3.906±0.010(b)	3.33±0.58(b)
	6	**-**	6.156±0.049(c)	6±1(c)
	6.5	**-**	7.896±0.015(d)	8±0(d)
	7	**-**	7.033±0.049(e)	5.33±0.58(e)
	7.5	**-**	6.896±0.015(e)	4.33±0.58(f)
	8	**-**	6.012±0.011(f)	4±0(f)
Additional carbon source	Starch	1	7.877±0.001(a)	8.67±0.58(a)
	Fructose	1	7.167±0.017(b)	7.67±0.58(b)
	Glucose	1	8.063±0.054(c)	10±1(c)
	Maltose	1	7.084±0.018(b)	8±1(a, b)
Additional nitrogen sources	Tryptone	0.3	8.073±0.004(a)	9.33±0.58(a)
	Yeast extract	0.3	8.430±0.010(a)	12±1(b)
	NH4NO3	0.3	8.066±0.005(a)	8±0.58(c)
Glucose concentration	Glucose	2	8.423±0.004(a)	10.33±0.58(a)
		4	8.743±0.004(a)	11±0(a)
		6	9.114±0.004(b)	12.33±0.58(b)
		8	9.742±0.001(b)	16±0(c)
		10	8.866±0.005(a)	11.33±0.58(d)
		12	8.805±0.004(a)	10.33±0.58(a)
Yeast extract concentration	0.1	-	8.410±0.010(a)	8.67±0.58(a)
	0.3	-	9.713±0.011(b)	11.33±0.58(b)
	0.5	-	9.065±0.004(b)	15.67±0.58(c)
	0.7	-	8.113±0.004(a)	13.67±0.58(d)
	0.9	-	7.205±0.004(c)	12.33±0.58(e)
Different metal ions	NaCl	0.05	10.134±0.056(a)	12±1(a)
	KCl	0.05	7.011±0.006(b)	8.67±0.58(b)
	MgCl2	0.05	5.706±0.009(c)	9.33±0.58(c)
	CaCl2	0.05	3.011±0.006(d)	10.33±0.58(d)
NaCl concentration		0.05	7.952±0.063(a)	11.33±.0.58(a)
		0.1	9.069±0.043(b)	14±0(b)
		0.2	8.006±0.004(a)	13.67±0.58(c)
		0.3	7.069±0.043(d)	12±1(a)

One-way ANOVA (Tukey’s Multiple Comparison test) was performed to check the potential statistical differences in case of the biomass (g/L) and antibacterial activity (ZOI-mm) in different fermentation conditions (incubation time, temperature, conc. of sugar and nitrogen sources etc.). There were valid statistical differences in most of the cases (P<0.05), the different letters a, b, c, d, e, and f indicates significant difference and same letter at two positions indicate no statistical differences.

Antibacterial activity related to fungal growth was measured at variable pH conditions of the medium (4–8). The optimum activity and growth were found at the slightly acidic pH of 6.5 (Table 2). Crossing or below this value the biomass production varies a little but potency to inhibit bacterial growth in-vitro changes a lot. This might be due to inhibition of fungal metabolic pathways at high acidic or basic condition that leads to inactivation of necessary enzymes or blocking of several intrinsic pathways. Fungi have the highest preference for carbohydrates as a source of carbon to obtain energy for normal growth and proper running of metabolic processes. The growth media was supplemented with additional carbon sources like glucose, fructose, maltose and starch. Starch supported the fungal growth and antibacterial production to greater extent than fructose or maltose but glucose is reported to be the best supplementary compound responsible for highest clear zone of inhibition against bacterial growth and maximum amount of biomass production. Glucose also being the cost effective one was selected over other carbon sources. The probable explanation of this carbohydrate-based growth and metabolism was due to varied effects of catabolic repression of different sugar sources on antibacterial production and biomass. After the selection of glucose as the most potent and economically feasible carbon source different concentrations of glucose (2 to 12 g/L) were used to evaluate the maximum response in terms of growth and metabolism for the production of antibacterial principles. It had been obtained from the study that 8 g/L of glucose had the highest influence on antibacterial (16 ± 0 mm) and biomass production (9.742 ± 0.001 g/L). Up-to a particular glucose concentration ranging from 2 g/L to 8 g/L the values increased but beyond that they decreased (Table 2). The upward and downward fluctuation from optimum glucose concentration (8 g/L) cause deterioration of both the parameters.

Nitrogen is known to be one of the best influencing factors for growth and metabolism of fungi. Here additional nitrogen sources were supplemented to the media to evaluate their effect on antibacterial performance and biomass production. It had been found that yeast extract had the highest influence (biomass 8.43 ± 0.010 g/L and zone of inhibition 12 ± 1mm) on fungal growth followed by tryptone (Organic nitrogen source) and NH4NO3 (inorganic nitrogen source). Different concentrations (0.1–0.9 g/L) of additional yeast extract were also used to detect the most effective concentration of yeast extract needed for the highest production of antibacterial compounds. The biomass and the antibacterial activity showed an upward movement but up-to certain limit then both the values started to fall below the optimum levels. *Colletotrichum alatae* LCS1 produced maximum amount of broad-spectrum antibacterial compounds and biomass at a concentration of 0.5 g/L yeast extract (Table 2).

Salts are generally known to influence the growth and metabolism of fungal strains. Metal ions act as catalyst or co-factors for the function of several key enzymes needed for production of growth necessary compounds. In this study NaCl supplemented media showed better production of antibacterial compounds than any other salt (KCl, CaCl2, and MgCl2) treated medium. As the concentration (0.05–0.3 g/L) of the salt increased in media the values got higher but after a certain level reaching the optimum position the values rapidly decreased (Table 2). A decrease in values might be due to accumulation of excess amount of metal ion leading to toxicity. Availability of oxygen in the growth medium had direct relation to organism’s growth and production of bioactive compounds. Generally, the Erlenmeyer flask was a closed system and the cotton plug act as a mild barrier between the external and internal system producing a micro environment but still air penetrates through it. The air slowly diffuses from head space to the liquid medium thus more the head space volume more the available oxygen on liquid medium. The increased medium volume lowers the surface area of the medium thus less space for interacting with air. Thus, the medium volume was negatively proportionate to factors like head space volume, interacting surface area and available oxygen i.e., more the medium volume less the head space volume and interacting surface area relating to less available oxygen on liquid medium and vice versa. But it is only involved in a positive relationship with medium depth i.e., higher medium volume; higher medium depth. In this experiment it has been documented that 50 mL of medium is suitable for optimum growth of the organisms and also for the production of antibacterial secondary metabolites (Table 3).

**Table 3:** Role of available oxygen on growth and antibacterial production by *Colletotrichum alatae* LCS1.

Medium volume (mL)	Total volume of flask (mL)	Head space volume (mL)	Medium depth (cm)	Surface area (cm)	Biomass (g/L)	Yield of bisabolol	Antibacterial activity (ZOI-mm)
25	320	295	1	2.79	4.763±0.215(a)	28.62 μg/mL(a)	6.33±0.58(a)
50	320	270	1.5	2.65	6.777±0.091(b)	42.46 μg/mL(b)	9.67±0.58(b)
75	320	245	2	2.53	5.112±0.415(c)	41.04 μg/mL(b)	9.33±0.58(b)
100	320	220	2.5	2.39	5.447±0.819(c)	20.34 μg/mL(c)	4.33±0.58(c)

One-way ANOVA (Tukey’s Multiple Comparison test) was performed to check the potential statistical differences between the data (column wise) of biomass (g/L), yield of bisabolol and antibacterial activity (ZOI- mm) in different medium volume (mL). There were valid statistical differences in most of the cases (P<0.05),the three different letters a, b, c indicates significant difference and same letter at two positions indicate no statistical differences.

#### Optimization by Box-Behnken design

One variable at a time (OVAT) methods have some limitations regarding the best suitable data interpretation among the different interacting factors but when it is coupled with RSM (Response surface methodology) it can minimize the process and hard-work generally interpreting the interaction between different factors. It not only reduces the effort but also provide a statistically significant model for proper optimization of fermentation procedures. Here a three level Box-Behnken design including four factors (Glucose concentration, yeast extract concentration, pH of medium, and fermentation time) with five replicates at the center point of each factor was introduced as a model for analysis of antibacterial compound production. The experimental values along with the predicted values by the statistical system are represented in Table 4. There was a variation in antibacterial production according to the variation of fermentation conditions. The replicated center points (five) always represented the highest values for antibacterial production. The predicted response (Y) for the production of antibacterial compounds (in terms of zone of inhibition) by the endophytic isolate *Colletotrichum alatae* LCS1 based on the coded fermentation factors is represented as the following equation: YAntibacterial activity (ZOI) = -194.97 + 87.64 X1+ 7.155 X2 + 161.09 M X3 492 + 7.61 X4- 88.90 X2–0.69777 X2–11.459 X2- 0.9800 X2–1.125 X1X2+ 0.050 X1X3+493 0.687 X1X4+ 0.075 X2X3 +0.8938 X2X4-0.3500 X3X4. Here Y(ZOI) was the predicted antibacterial activity and X1, X2, X3, X4 were coded factors of yeast extract concentrations, glucose concentrations, medium pH and fermentation time respectively.

**Table 4:** Experimental design and results of the Box-Behnken design for the optimization of the antibacterial activity of the fungal isolate *Colletotrichum alatae* LCS1.

**Run**	**Independent variables**				**Response**	
	X1: YEC (g/L)	X2: GC (g/L)	X3: M pH	X4: FT (day)	Measured	Predicted
1	0 (0.5)	0 (8)	0 (6.5)	0 (6)	22.4000 (a1)	22.4580 (a1)
2	1 (0.7)	0	1(7)	0	15.5100 (a2)	15.5600 (a2)
3	0	0	-1 (6)	-1(4)	17.3900 (a3)	17.3529 (a3)
4	0	1 (10)	-1	0	15.5700 (a4)	15.6404 (a4)
5	-1 (0.3)	0	0	1 (8)	14.6800 (a5)	14.7471 (a5)
6	0	-1 (6)	1	0	18.3500 (a6)	18.3488 (a6)
7	0	1	0	1	14.2000 (a7)	14.1783 (a7)
8	0	0	-1	1	15.0400 (a8)	14.9996 (a8)
9	-1	0	1	0	17.5400 (a9)	17.5083 (a9)
10	0	1	0	-1	14.5500 (a10)	14.5167 (a10)
11	0	0	0	0	22.5200 (a11)	22.4580 (a11)
12	-1	-1	0	0	18.5000 (a12)	18.4646 (a12)
13	0	0	1	1	14.4500 (a13)	14.4013 (a13)
14	0	1	1	0	15.3800 (a14)	15.4571 (a14)
15	1	0	0	1	13.5700 (a15)	13.5638 (a15)
16	0	-1	-1	0	18.7700 (a16)	18.7621 (a16)
17	1	0	0	-1	15.4400 (a17)	15.4421 (a17)
18	1	-1	0	0	17.6000 (a18)	17.5563 (a18)
19	0	0	1	-1	17.4000 (a19)	17.3546 (a19)
20	-1	0	-1	0	17.8500 (a20)	17.8167 (a20)
21	0	0	0	0	22.5400 (a21)	22.4580 (a21)
22	1	0	-1	0	15.8000 (a22)	15.8483 (a22)
23	-1	1	0	0	16.5500 (a23)	16.5079 (a23)
24	0	-1	0	-1	19.8000 (a24)	19.8383 (a24)
25	-1	0	0	-1	18.1000 (a25)	18.1754 (a25)
26	1	1	0	0	13.5500 (a26)	13.4996 (a26)
27	0	-1	0	1	14.8200 (a27)	14.8700 (a27)
28	0	0	0	0	22.4100 (a28)	22.4580 (a28)
29	0	0	0	0	22.4200 (a29)	22.4580 (a29)

One-way ANOVA (Tukey’s Multiple Comparison test) was performed to check the potential statistical differences (P<0.05) between the measured and predicted antibacterial action (zone of inhibition). There were no statistical differences between each data set (row wise) and similar letters (a1-a29) in each row indicates the data are same and lacks statistical differences.

**Fig 5:**
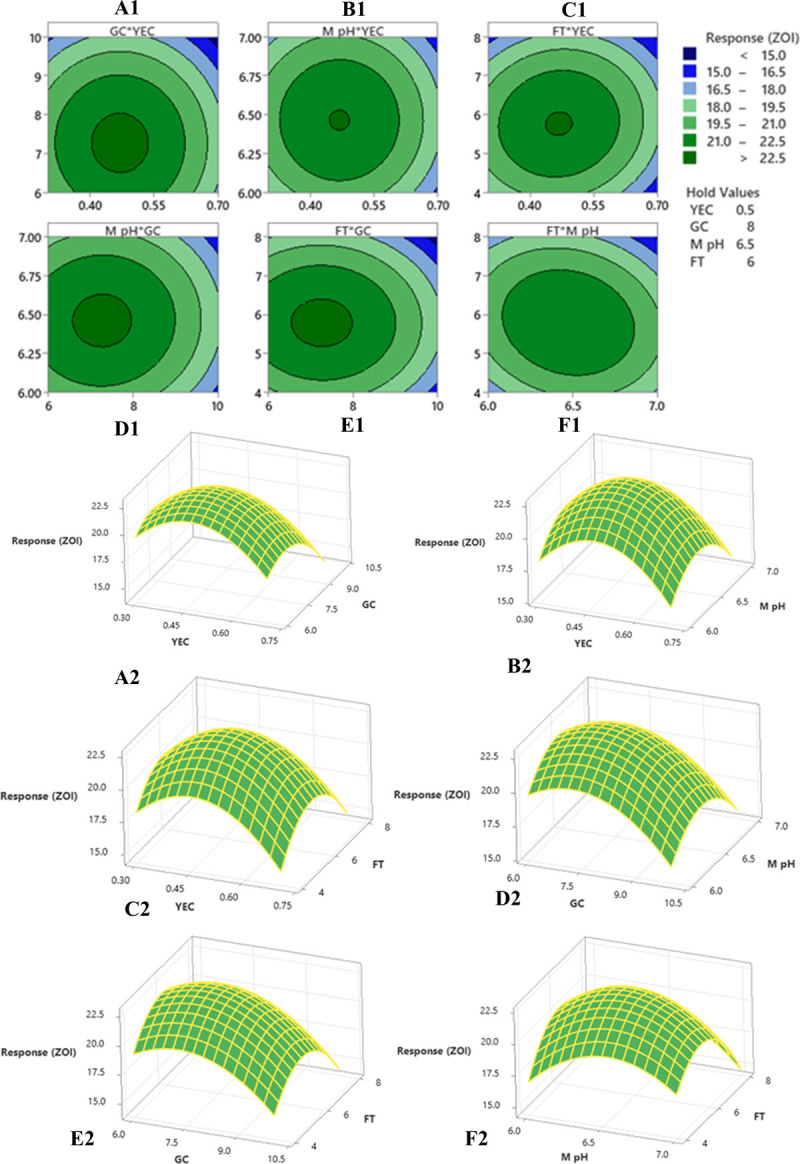
The contour plot and 3D-plot with 2D-projection showing the most important interactions of factors in RSM optimization of antibacterial activity by *Colletotrichum alatae* LCS1 (A1 &A2) between yeast extract conc. (YEC) vs. glucose conc. (GC) at fermentation time (FT) 7 days and medium pH (M-pH) 6.5 (B1 & B2) between YEC vs. M-pH at FT 7 days and GC 8 (C1 & C2) between YEC vs. FT at GC 8 and M-pH 6.5 (D1 & D2) between GC and M-pH at FT 7 days and YEC of 0.47 (E1 & E2) between GC vs. FT at YEC 0.47 and M-pH 6.5 (F1 & F2) between M-pH vs. FT at GC 8 and YEC 0.47.

A regression analysis was recommended for checking the goodness of fit of the response surface methodology (RSM) with experimental output data. The F test data having a large value of 3979.93 indicated that the model was significant. There is no chance that this type of large F value could originate due to noise issues. The adjusted determinant co-efficient (R^2^) of the model help in evaluation of the goodness-of-fit of the regression equation. R^2^ value was found to be 0.9999 which denotes that there was a high degree of correlation between the experimental and predicted value. It could be concluded that the fitness of the model was good as it has the lack of fit F value of 1.09 which was not at all significant related to the obtained pure error. The model P value being less than 0.0001 focus on the fact that the model equation was appropriate regarding the antibacterial production. The P value for lack of fit for this polynomial equation was 0.509 which is higher than 0.05 that indicates the accuracy of this system. The linear and quadratic effects of glucose concentration, yeast extract concentration, fermentation time and pH of fermentation medium were significant (P<0.0001) in this model (Table 5). The interactions among the four different variables regarding the production of antibacterial compounds were analyzed. The interaction where P<0.0001, they have positive effects where-as the interaction between medium pH and glucose concentration, medium pH and yeast extract concentration (P˃0.0001) were of least importance as the P values were greater than 0.0001. Response surface plots were constructed using contour plotting through the help of Minitab. The contour plots focused on the portions where the best interactions had been made between the different interacting factors to obtain the optimum results (Fig 5). The model assumed a maximum response for antibacterial production in terms of zone of inhibition of 22.74 mm when the four interacting factors will be YEC (yeast extract concentration) 0.47 g/L, GC (glucose concentration) 7.53 g/L, MpH (medium pH) 6.46 and FT (fermentation time) 5.61 days (134 hours). The predictions were performed in laboratory to evaluate its success and it had been found that following the parameters *Colletotrichum alatae* LCS1 yielded a response of 22.66 ± 0.894 mm zone of inhibition. Experimental verification established the fitness of the model and also the optimum conditions were finally shorted out through the minimum effort.

**Table 5:** ANOVA for response surface quadratic regression model of antibacterial productions by endophytic *Colletotrichum alatae* LCS1.

Source	DF	Adj SS	Adj MS	F-Value	P-Value
Model	14	231.289	15.6709	3979.93	0.000
Linear	4	59.097	17.0034	3178.69	0.000
YEC (X1)	1	13.95	13.6054	2767.99	0.000
GC (X2)	1	31.012	29.0765	5457.79	0.000
M pH (X3)	1	0.391	0.2670	58.88	0.000
FT (X4)	1	27.398	23.1205	4443.60	0.000
Square	4	174.876	43.3433	8198.94	0.000
2) YEC*YEC (X1	1	69.768	65.7646	14762.66	0.000
2) GC*GC (X2	1	53.147	51.5307	10997.42	0.000
2) MpH*MpH (X3	1	49.876	43.4540	9547.89	0.000
2) FT*FT (X4	1	98.768	95.457	19987.78	0.000
2-Way Interaction	6	9.690	1.2986	276.57	0.000
YEC*GC (X1X2)	1	1.208	1.1092	241.99	0.000
YEC*M Ph (X1X3)	1	0.001	0.0001	0.02	0.887
YEC*FT (X1X4)	1	0.707	0.7005	198.72	0.000
GC*M Ph (X2X3)	1	0.019	0.0123	3.89	0.119
GC*FT (X2X4)	1	6.387	6.3793	1139.88	0.000
M pH*FT (X3X4)	1	0.091	0.0989	20.00	0.001
Error	14	0.067	0.0069		
Lack-of-Fit	10	0.096	0.0041	1.10	0.505
Pure Error	4	0.017	0.0057		
Total	28				
R-sq		99.96%			
R-sq(adj)		99.95%			
R-sq(pred)		99.89%			

### Yield of bisabolol

Culture filtrates of LCS1 containing bisabolol produced at different phases of optimization were purified and compared with data obtained from standard curve provided by standard bisabolol. From the standard curve, the occurrence of bisabolol in culture filtrates are found to be 41.42 μg/mL before optimization (10 mm clear zone of inhibition), after OVAT optimization 61.54 μg/mL (16 mm), after OVAT+RSM 83.60 μg/mL (22.66 mm). Bisabolol production was elevated up-to 2-fold when all the parameters were in optimum state. The enhancement of bisabolol production is confirmed by comparing the area percentage and determining the concentration of bisabolol from calibration curve (S2 Fig). It has been found that the area percentage of the preoptimized and post-optimized bisabolol is almost double and that supports the enhanced conc. of bisabolol in the system.

### Purification and characterization of the active compound

#### TLC and bioautography

**Fig 6:**
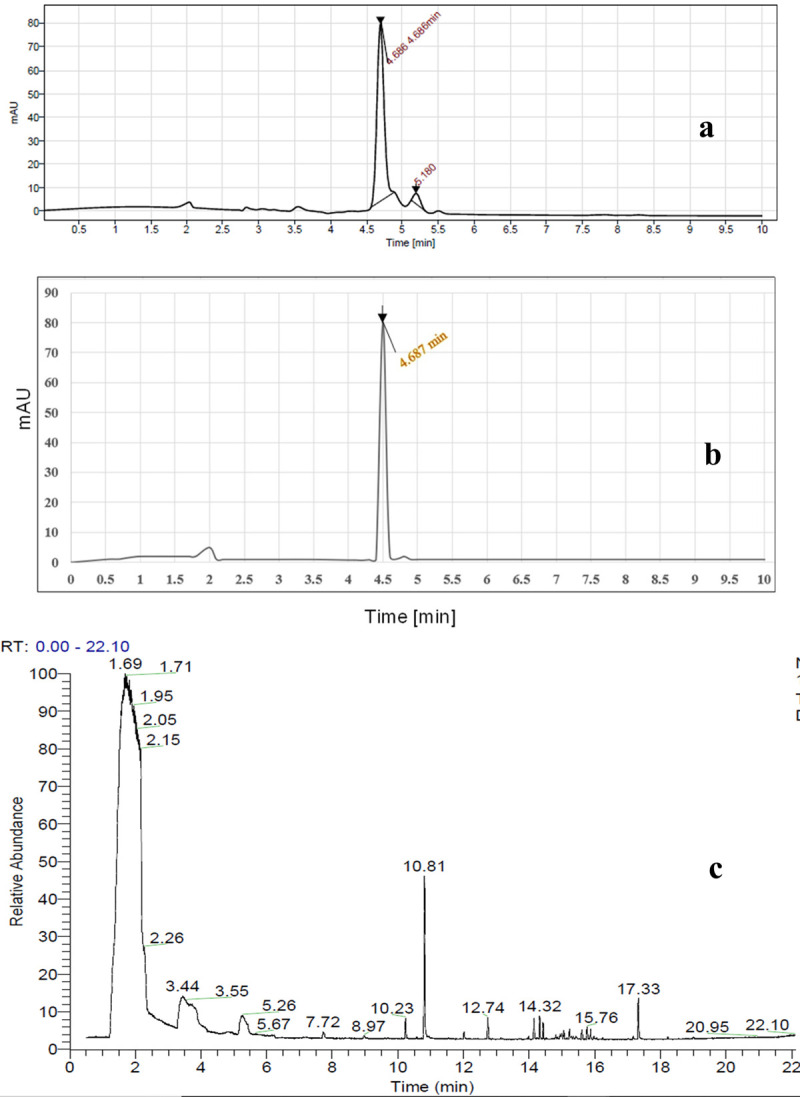
a HPLC chromatogram of antibacterial fraction of LCS1 extract obtained using a C18 reverse phase column and gradient elution was used with the mobile phase composed of (A) acetonitrile–water–phosphoric acid (19:80:1) and (B) acetonitrile with a flow-rate of 0.8 mL/min. Fig 6B HPLC chromatogram of standard bisabolol chemical Fig 6C Full scan chromatographic profile obtained from the bioactive fraction (antibacterial) of endophytic fungi *Colletotrichum alatae* LCS1.

The principal compounds were separated in a 9.1:0.9 ratio of polar-nonpolar (methanol-chloroform) solvent and bands were visible upon UV exposure. The band with Rf value of 0.67 showed the highest zone of inhibition after TTC treatment against bacterial pathogens (Fig 2B and 2C). The constituents of that band had broad spectrum antimicrobial activity against Gram positive and Gram-negative bacterial pathogens and that bioactive fraction was selected for GC-MS analysis. The purified extract with only bisabolol as the constituent is run on HPLC system and the single peak chromatogram is obtained (Fig 6A) and confirmed with the pure bisabolol HPLC spectra (Fig 6B).

GC-MS of the active fraction. Following the results of TLC analysis and bioautography the bioactive compounds were subjected to GC- MS analysis. In total 17 compounds were identified by the NIST library and they were recorded with molecular weight, retention time of detection and area percentage in the Table 6 along with their previously known bioactivities (chemical structures are presented in S3 Fig). The compounds were amino alcohol, phenols, organic acids (oxalic acid, and butyric acid (a type of carboxylic acid), pterin-6-carboxylic acid), sesquiterpenes (cedrane and longipinene), monoamine alkaloids (cathinone), and dimethylamine etc. Insect repellent compound naphthalene (an aromatic hydrocarbon) is known to be produced at a retention time of 17.33 min (Fig 6C). Abundance of long chain hydrocarbons like 4-amino-1-pentanol, 3-methyl 1-butanol (isopentyl alcohol), d-alaninol confirms the occurrence of myco-diesel like compounds and also ensures the fuel producing abilities of the endophytic *Colletotrichum alatae* LCS1. The EI mass spectrum of LCS1 derived bisabolol and standard bisabolol is compared to confirm its occurrence (S4 Fig). It produces an essential oil bisabolol (a monocyclic sesquiterpene alcohol) which is of immense industrial importance due to its multipurpose use in food industry as flavour enhancer, in pharmaceutical industry as ingredients of aroma therapies and as anti-irritant, anti-inflammatory, anti-microbial compound combined with skin healing properties [18]. Alpha bisabolol not only inhibits bacterial growth but also increases the penetrance of other antimicrobial agents through the cell wall (cause leakage of DNA and protein contents) and increases the antibacterial effect synergistically acting with other bioactive secondary metabolites [36]. Compounds like Oxalic acid, Butyric acid, 2-methyl-, ethyl ester, Pthalic acid, 7-isopropyl-1-methyl phenanthrene, cedrene were previously reported to exhibit broad spectrum antibacterial activity against a number of potent human pathogenic microorganisms like *Pseudomonas aeruginosa*, *Bacillus melitensis*, *Salmonella typhi*, *Staphylococcus infantis*, *Salmonella paratyphi*, *Escherichia coli*, *Streptococcus faecalis*, *Staphylococcus epidermidis*, *Salmonella typhimurium*, *Clostridium perfringens*, *Listeria monocytogenes*, *Staphylococcus aureus*, *Bacillus subtilis*, *Solobacterium moorei*, *Streptococcus pyogenes* respectively. Alpha bisabolol is effective against human dermatophytes and phytopathogenic fungi *Trichophyton tonsurans*, *T*. *mentogrophytes*, *T*. *rubrum*, *Microsporum canis*, *Aspergillus flavus*, *Aspergillus fumigatus*, *Aspergillus niger*, *Aspergillus terreus*, *Fusarium oxysporum*, *Fusarium solani*, and *Fusarium verticillioides* [37]. It has also been characterized as anti-*Leishmania amazonensis* and a potent agent to cope up with leishmaniasis [38]. Earlier bisabolol has been reported from *Myoporum grassifolium*, *Matricaria chamomilla*, *Vanillosmopsis arborea*, *Eremanthus erythropappus*, *Salvia runcinata*, *Smyrniopsis aucheri* [18,19,39]. It is the first time an endophytic fungi *Colletotrichum alatae* LCS1 isolated from *Lycopodium clavatum* collected from an unpopular uncommon geographical place produced this essential oil in a good amount.

**Table 6:** List of bioactive compounds produced by *Colletotrichum alatae* LCS1and their respective bioactivities.

Sl. No.	Name of the compound	RT (min)	Area %	Ch. formula	MW (g mol^-1^)	Biological activity
1	4-Amino-1-pentanol (amino alcohol)	2.26	17.64	C5H13NO	103	------
2	3-methyl 1-butanol (Isopentyl alcohol)	3.44	9.15	C5H12O	88	Antimicrobial activity against phytopathogens [40]
3	Butyric acid, 2-methyl-, ethyl ester	5.26	5.22	C7H14O2	130	Broad spectrum Antibacterial activity against poultry pathogens *Salmonella typhimurium* and *Clostridium perfringens* [41]
4	2-(2-Aminopropyl) phenol	6.16	3.92	C9H13NO	151	Antibacterial
5	Pthalic acid	7.72	2.61	C8H604	166.14	Broad spectrum antibacterial activity against 10 pathogenic microorganisms [42]
6	d-Alaninol (amino alcohols)	8.12	1.30	C3H9NO	75	------
7	7-isopropyl-1-methyl phenanthrene	8.40	1.30	C18H18	234	Broad spectrum antibacterial activity [43]
8	p-Hydroxyphenylacetic acid	8.99	1.30	C8H8O3	152.15	Broad spectrum antibacterial activity against *L*. *monocytogenes*, *S*. *aureus*, *P*. *aeruginosa*, *E*. *coli*, *C*. *albicans* [44]
9	Cathinone or Norephedrone	10.23	5.22	C9H11NO	149	----
10	à-Bisabolol (sesquiterpene)	10.81	30.71	C15H26O	222	Antibacterial activity against halitosis pathogen *Solobacterium moorei* [36]
11	Longipinine (sesquiterpene)	12.01	1.30	C15H24	204.35	----
12	2-amino-1-(4-methyl phenyl) propane	12.74	5.22	C10H15N	149	-----
13	Cedrene (sesquiterpene)	14.15	5.22	C15H24	204	Broad spectrum antimicrobial activity against human pathogenic microbes (*Staphylococcus aureus* ATCC 6538, *Pseudomonas aeruginosa* ATCC 27853 and *Candida albicans*) [45]
14	Naphthalene	17.33	8.49	C10H8	128	Antimicrobial and insecticidal property [46]
15	Pterin-6-carboxylic acid	19.00	1.30	C7H5N5O3	207	Antimicrobial and antioxidant activities of phenolic acid [47]
16	Dimethylamine	20.95	1.30	C2H7N	45	Broad spectrum antimicrobial activity against *S*. *aureus* and *E*. *coli* [48]
17	Oxalic acid	22.10	1.30	C2H2O4	90	Broad spectrum antibacterial activity against plant and human pathogenic microorganisms. [49]

### Antioxidative potency of the endophytic culture extracts

**Fig 7:**
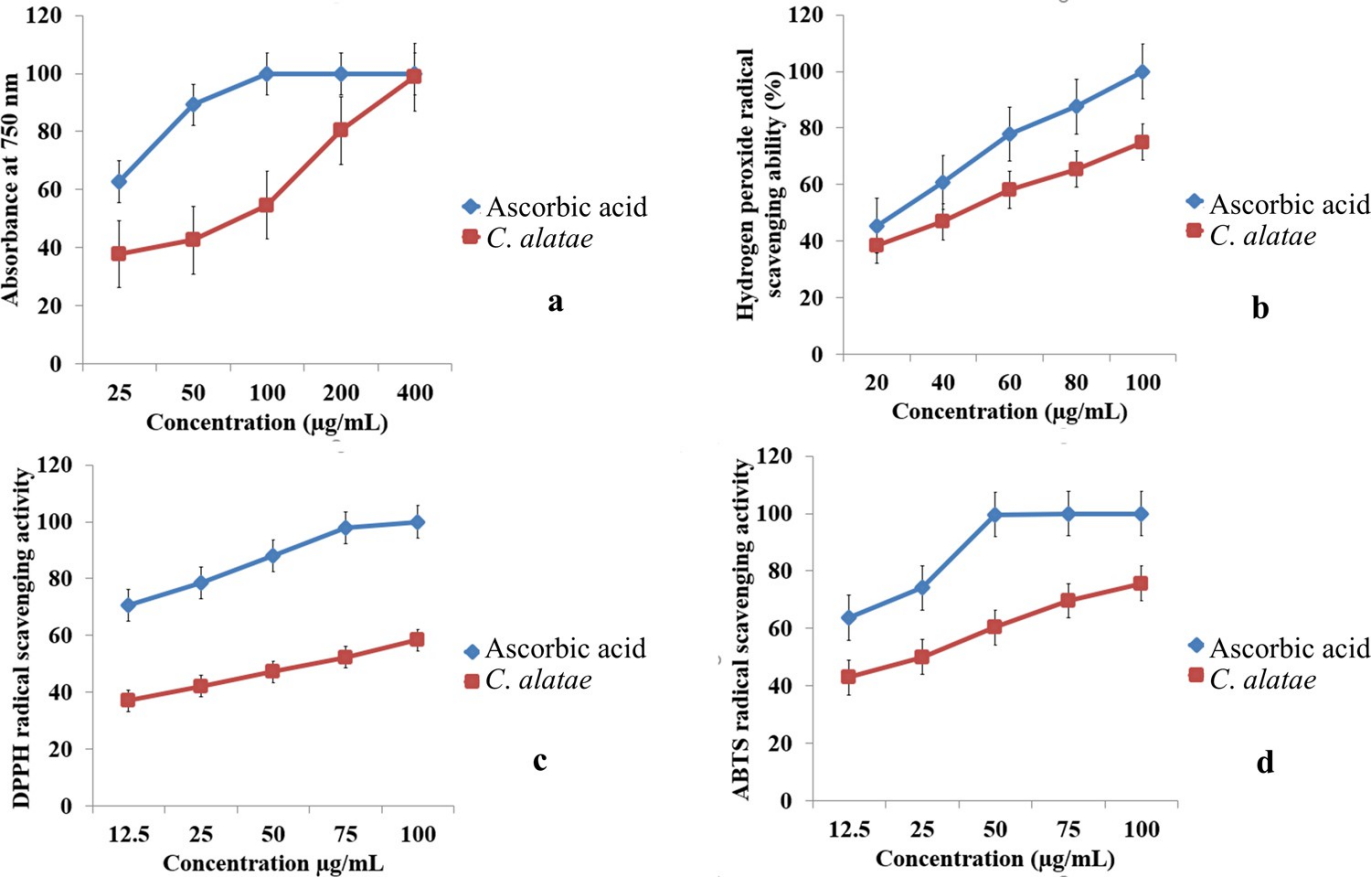
Antioxidant activity of endophytic culture extract (ethyl acetate fraction) of *Colletotrichum alatae* LCS1 and ascorbic acid as standard. a–Ferric reducing antioxidant power assay. b- Hydrogen peroxide radical scavenging ability. c- DPPH radical scavenging activity. d–ABTS radical scavenging assay Values on the graphs are the means ± Standard error (SE) of the three replicates.

Bioactive principles of endophytic fungi are proven to be the source of potent antioxidative compounds [50]. The EA extracts of six isolated endophytic fungi were tested for their free radical scavenging activity by colorimetric DPPH assay and *Colletotrichum alatae* LCS1 is proved to be the most potent producer. Colour change of methanolic DPPH from deep purple to light yellow is the indication of radical scavenging ability of the endophytic culture extract. Ethyl acetate fraction of the isolate *Colletotrichum alatae* LCS1 being the most potent antioxidant producer (confirmed using DPPH assay) was screened for its ability to scavenge other free radicals like ABTS and hydrogen peroxide. IC50 values were determined by preparing different concentrations of culture extract and adding them to the respective free radical generator. The IC50 values of the culture extract against different free radical 594 producers were reported as 52.75 ± 5.47, 56.37 ± 6.06, 23.38 ± 5.32, and 82.87 ± 6.47 μg/mL for scavenging ability of H2O2 radicals, DPPH radicals, ABTS radicals and FRAP (Ferric reducing antioxidant power) respectively. In each case there was a statistical difference between the values of standard (ascorbic acid) and culture extract (Table 7). The standard curves are prepared for both the ascorbic acid standard and culture extract for four separate cases (Fig 7). According to the efficiency of the culture extract to fix free radicals depending on the IC50 values they could be assigned in an order of scavenging ABTS ˃ H2O2 ˃ DPPH ˃ FRAP radicals.

**Table 7:** Antioxidant activity of *Colletotrichum alatae* LCS1 methanolic culture extract isolated from *Lycopodium clavatum*.

	Antioxidant assays	*Colletotrichum alatae*	Standard
**IC50**	Scavenging ability of hydrogen peroxide	52.75 ± 5.47^a^	12.03 ± 2.21^b^
	Scavenging ability of DPPH radicals	56.37 ± 6.06^a^	12.80 ± 1.73^b^
**value**			
**(μg/m**	Scavenging ability of ABTS radicals	23.38 ± 5.32^a^	6.02 ± 2.62^b^
**L)**			
	Ferric reducing antioxidant power	82.87 ± 6.47^a^	26.26 ± 4.96^b^

One-way ANOVA (Tukey’s Multiple Comparison test) was performed to check the potential statistical differences and the standard (ascorbic acid) showed statistically valid differences (P<0.05, the two different letters a and b in each case indicates significance differences) from the *Colletotrichum alatae* LCS1 extract.

## Discussion

Antimicrobial resistance has become a serious global concern and it is high-time to adopt novel strategies to overcome this situation [51]. Bioactive compounds from endophytic communities have just opened a new sector in this respect [52,53]. Endophytes are all square in action and in our present study we have elucidated the antibacterial and antioxidative action of endophyte derived bioactive molecules against a variety of pathogenic microorganisms. An age old traditional ayurvedic herb *Lycopodium clavatum* was selected for endophyte isolation which bears enormous ethnomedicinal importance e.g.- wound healing, skin soothing and hepatoprotective [54–56].

Isolate LCS1 stores some amount of antibacterial principles with in their mycelial biomass but the maximum quantity of antibacterial compounds are present on the extracellular environment. Similar types of findings were made by [22] where endophytic isolates produce a minute number of antibacterial metabolites with in the mycelial cells but the majority of components were present at the cell free culture extract. Another recent example from the work of [57] supports our present investigational outcome where they isolated bioactive compounds from fungal endophytes of *Pelargonium sidoides* with potent antibacterial activity against clinically important bacterial strains including MRSA and other enteric disease-causing agents.

Solvents play a major role on extracting bioactive compounds from culture broth, it depends on the compatibility of the solvent, components of secondary metabolites and also polar non polar interaction. Ethyl acetate is proved to be the most potent extracting agent in comparison to other three solvents as it is the universal solvent used for extraction of components of biotechnological importance. Other solvents like ethyl-ether, methanol and methanol-water in a proportion of 1:1 was not the efficient most in this purpose yielding a very high amount of MIC values against Gram positive pathogens *Bacillus subtilis* and *Bacillus cereus*. Findings on antibacterial principles extracted from olive tree endophytes using ethyl acetate as the efficient solvent supports our present outcome [22]. Other investigation lead by [58,59] also reported highest antimicrobial activity of the ethyl acetate extract over other agents.

The host plant extract itself is known to be moderately antibacterial and it can help in slowing down the growth of bacterial pathogens acting in a static mode of action. Lycovatine alkaloid from *L*. *clavatum* var *robustum* was antifungal towards *Cryptococcus neoformans* and *Aspergillus niger* at MIC values of 0.52 μg/mL and 2.05 μg/mL [60]. The host plant is known to harbour bioactive alkaloids named clavatine, clavatoxine, and clavolonine, lycopodine [61,62]. Experimental outcomes revealed that when bacterial pathogens are grown on a nutrient broth medium previously supplemented with host plant extract the antibacterial activity of the endophytic culture extract got enhanced even to double than the previous results for some selected pathogens. These types of findings are of high industrial or commercial values as it may scale up the production of antimicrobial compounds from some selected species of endophytes by prior addition of some inducing agents. The possible explanation of this incident could be due to synergistic effect of both the bioactive secondary metabolites produced by the plant (alkaloids) and endophytes have restricted the pathogenic growth. Enhancement of antibacterial activity of the endophytic isolates in case of pre-supplemented plant extract containing medium has been reported [12,22]. Production of anti-candidal secondary bioactive metabolites by *Phomopsis* sp.; an endophyte of medicinal herb *Orthosiphon stamineus* got enhanced upon addition of aqueous host extract [63].

EA culture extract exhibited a strong cidal action against Gram positive pathogens than the Gram negative one, evidenced by the quantitative measurement of macromolecules (DNA and protein) on the extracellular environment released due to leakage of cell wall components. The amount of DNA and protein got drastically increased upon increase of the treatment time. This focuses on another fact that more time the active principles obtain to interact with the target cells the more they cleave up the cell wall and cause lethal leakage of cell contents. Recent investigations on antibacterial principles of *Alternaria alternata*, an endophyte of *Azadirachta indica* revealed similar expression [43]. The penetration of different types of antibacterial compound is mediated by the occurrence of high percentage of essential oil bisabolol. It not only directly interacts with bacterial cell but also cause increased penetration of antibacterial secondary metabolites [36].

The most efficient finding of this study is the inhibition of MRSA growth. Staphylococci are non-capsulated, perfectly spherical shaped cocci occurring in a bunch of grapes like appearance and especially *S*. *aureus* are extremely fatal upon penetration to skin causing infection in bones, joints, bloodstreams, heart valves, lungs etc. [23]. The probable cause of success of the bioactive metabolites to restrict MRSA growth is probably due to its penetration to cell wall as an action of bisabolol.

In killing kinetics experiment all the tested pathogens at MIC*0 showed normal lag to stationary phase of the growth and a mild static effect of bacterial culture was seen in MIC/2 that indicates the strong antibacterial nature of the component. In MIC values the CFUs reduced remarkably after 12 h and entered in to stationary phase of growth. At MBC (MIC*2) viable cell counts fall drastically and at 12 h or 24 h stage their growth ceased totally. The most efficient cidal action was found at concentrations four times higher than the MIC values and viable cell counts falls sharply to zero after 6 h of continuous treatment. In, all the cases there were a 3 Log CFU/mL reduction in cell number indicating the cidal activity of the antibacterial components.

Microbial fermentation is highly influenced by the media compositions, concentration of essential macro and micro nutrients, medium pH, incubation time and temperature etc. which are directly proportional to qualitative and quantitative production of secondary metabolites [64]. Here for antibacterial optimization, OVAT (One variable at a time) approach has been adopted and parameters like carbon source, nitrogen source, fermentation time, fermentation temperature, medium pH, salt, medium volume-oxygen availability was optimized (Tables 2 and 3). Out of this tested parameter the final parameters were found to be; glucose (7.53 g/L) as carbon source, yeast extract (0.47 g/L) as nitrogen source, NaCl (0.10 g/L) as salt, 6.46 as medium pH, fermentation time-5.61 days (134 h), 26°C as incubation temperature. For Response surface methodology (Box Behnken design), four combinatorial factors had been checked for their together influence on highest antibacterial production (prime component bisabolol) against the potent antibiotic resistant strain MRSA. Endophytic microbiota showing highest response in presence of glucose as prime carbon source, yeast extract as the organic nitrogen source and vitamin precursors has been previously reported by [21]. Increase in glucose concentrations cause a rapid increase in fungal biomass but stress is directly proportional to production of secondary metabolites. So, after a certain level the increase in glucose concentration cause decrease in antibacterial production as the fermentation situation is not facing stress issues and glucose may block some necessary enzymes needed for bisabolol production.

Response surface methodology using OVAT method had been adopted by several workers finding the maximum production of antibacterial compounds fixing the fermentation parameters suitable for the organism [65]. Antimicrobial activity of the endophytic fungi *Aspergillus* sp. Cpr5 isolated from *Calotropis procera* root was enhanced using varying fermentation compositions [66]. Scaling up of antibiotics production from actinobacterial (*Streptomyces* sp. JAJ06 from Indian coastal solar saltern, *Streptomyces afghaniensis* from marine ecosystem, *Streptomyces rimosus* AG-P1441 and Cassava rhizosperic *Streptomyces* sp. 1–14) sources are popular patterns for fermentation technology to achieve economically feasible and efficient situations [67–69]. *Xenorhabdus bovienii* and *Bacillus subtilis* AD35 were optimized using response surface methodology and antibiotic productions (xenortides, xenematide, xenoxoumacin, xenorhabdins, xenocoumacin, nematophin and di-(2-ethylhexyl) phthalate (DEHP) respectively) were enhanced remarkably [70,71].

*Colletotrichum alatae* LCS1 of Lycopodium origin produces the unique essential oil bisabolol which is purified on silica glass column and the purity is checked using HPLC and GC-MS chromatogram and that purified bisabolol is tested each time for its antibacterial production ability during the optimisation studies and it has been noticed that bisabolol along with other 16 bioactive compounds show a slightly higher clear zone of inhibition (2.5 ± 0.5 mm) than the purified bisabolol. So, it could be drawn to discussion that endophytic microflora produces plethora of components and works in a cumulative manner for enhancement of bioactivity. Here, in this study the bisabolol production has been optimized by response surface methodologies and OVAT. It has been obtained from the results that bisabolol production shows a two-fold increase than the pre-optimised one (41.42 to 716 83.60 μg/mL).

Oxidative stress may lead to neuro degenerative diseases, brain dysfunction, fall of immune system, and cancer etc. The one and only solution is consumption of antioxidants in daily diet but synthetic ones (like butylated hydroxyanisol and butylated hydroxytoluene) are causing toxicity issues leading to search for the novel and safe one from natural sources [72,73]. Endophytic fungi are store house of antioxidative metabolites having direct effect on protection of cells from oxidative damages [74]. In the present study isolate LCS1 is proved to be potent radical scavenger with low IC50 value indicating the higher chances of its pharmaceutical or clinical acceptance. Comparative data with potent standard ascorbic acid was made and the isolate was reported to be nearly efficient to ascorbic acid. Bisabolol is popular for its antioxidant properties too [75]. It interferes with ROS (reactive oxygen species) inhibiting the luminol-amplified chemiluminescence at low values for *C*. *albicans* and N-formyl-methionyl-leucyl-phenyl-alanine respectively improving the antioxidant capacity [76]. Occurrence of this sesquiterpene as a major component was the prime cause of good antioxidative activity exhibited by the culture filtrates of the isolate. Terpenes (bisabolol, cedrene, longi-pinene) act on the chain reactions necessary for free radical generation and convert them into inert components by irreversible oxidation [77]. In this study endophytic *C*. *alatae* LCS1 from *L*. *clavatum* source represents a variety of bioactive molecules with both antibacterial as well as antioxidative activity at proper optimized fermentation parameters.

## Supporting information

S1 Fig Plate (a, c, e, g, i) and microscopic morphology (b, d, f, h, j) of *Lycopodium clavatum* endophytes a, b-*Scopulariopsis* sp. c, d-*Lasidiplodia* sp. e, f- *Pestalotiopsis* sp., g, h- *Phomopsis* sp. i, j- *Phoma* sp.
(DOCX)

S2 Fig Standard curve of bisabolol. (DOCX)

S3 Fig Chemical structures of the bioactive compounds derived from NIST library. (DOCX)

S4 Fig a Electron-ionisation mass spectrum of bisabolol obtained from LCS1 extract. b Electron-ionisation mass spectrum of standard bisabolol.
(DOCX)

S1 Table a. Antibacterial activity (in terms of clear zone of inhibition in mm) of the endophytic isolates against pathogenic bacteria. b MIC values (μg/mL) of culture extract and mycelial extract of *Colletotrichum alatae* LCS1 obtained using two different types of solvent.
(DOCX)
