# Supplementary figures and images for: Bioactivity study and metabolic profiling of Colletotrichum alatae LCS1, an endophyte of club moss Lycopodium clavatum L
Source: PLoS One. 2022 Apr 28;17(4):e0267302. doi: 10.1371/journal.pone.0267302 (PMC9049576; doi:10.1371/journal.pone.0267302)

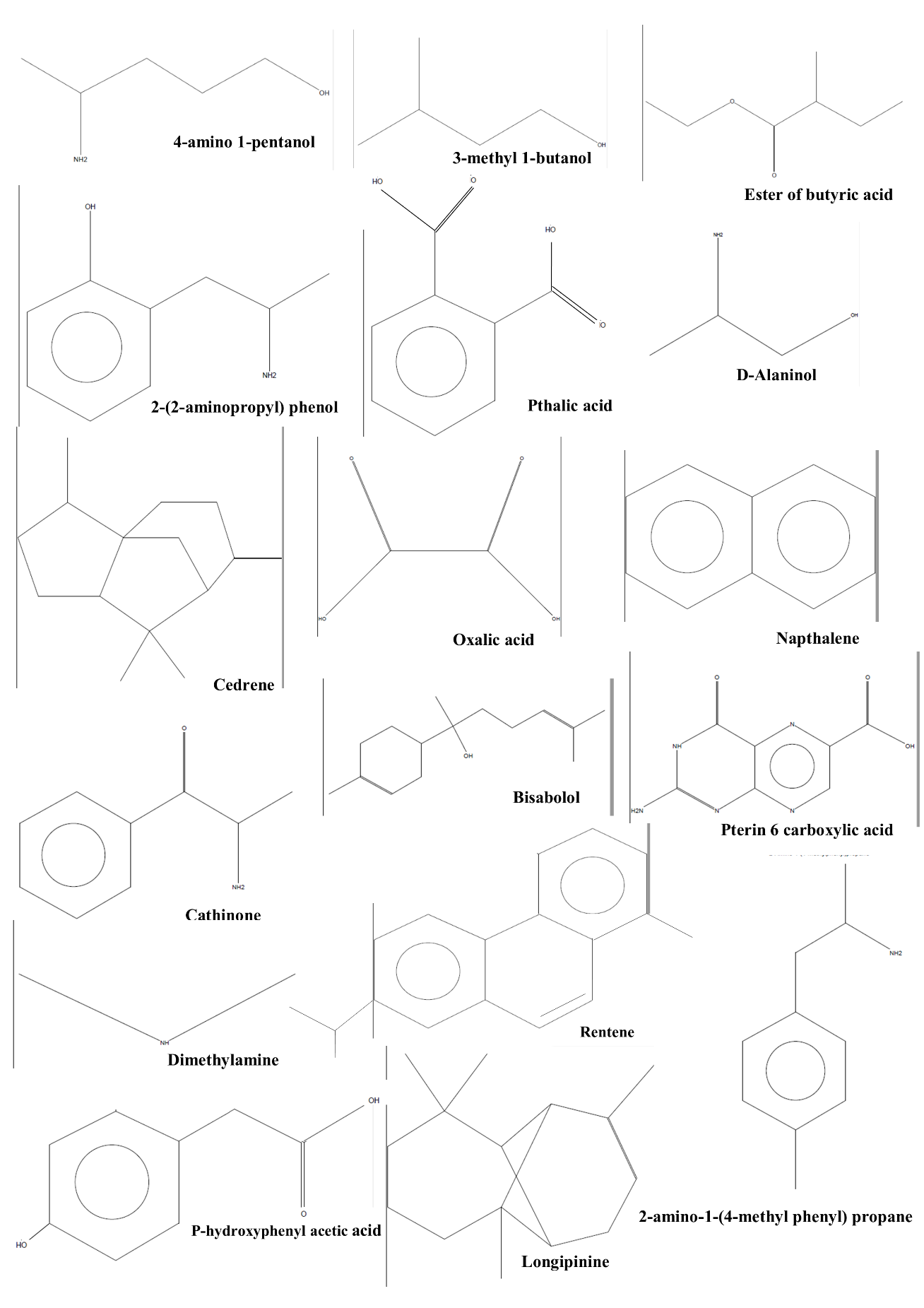

Supplement: S1 Fig — Plate (a, c, e, g, i) and microscopic morphology (b, d, f, h, j) of Lycopodium clavatum endophytes a, b-Scopulariopsis sp. c, d-Lasidiplodia sp. e, f- Pestalotiopsis sp., g, h- Phomopsis sp. i, j- Phoma sp. (DOCX) [file pone.0267302.s001.docx]

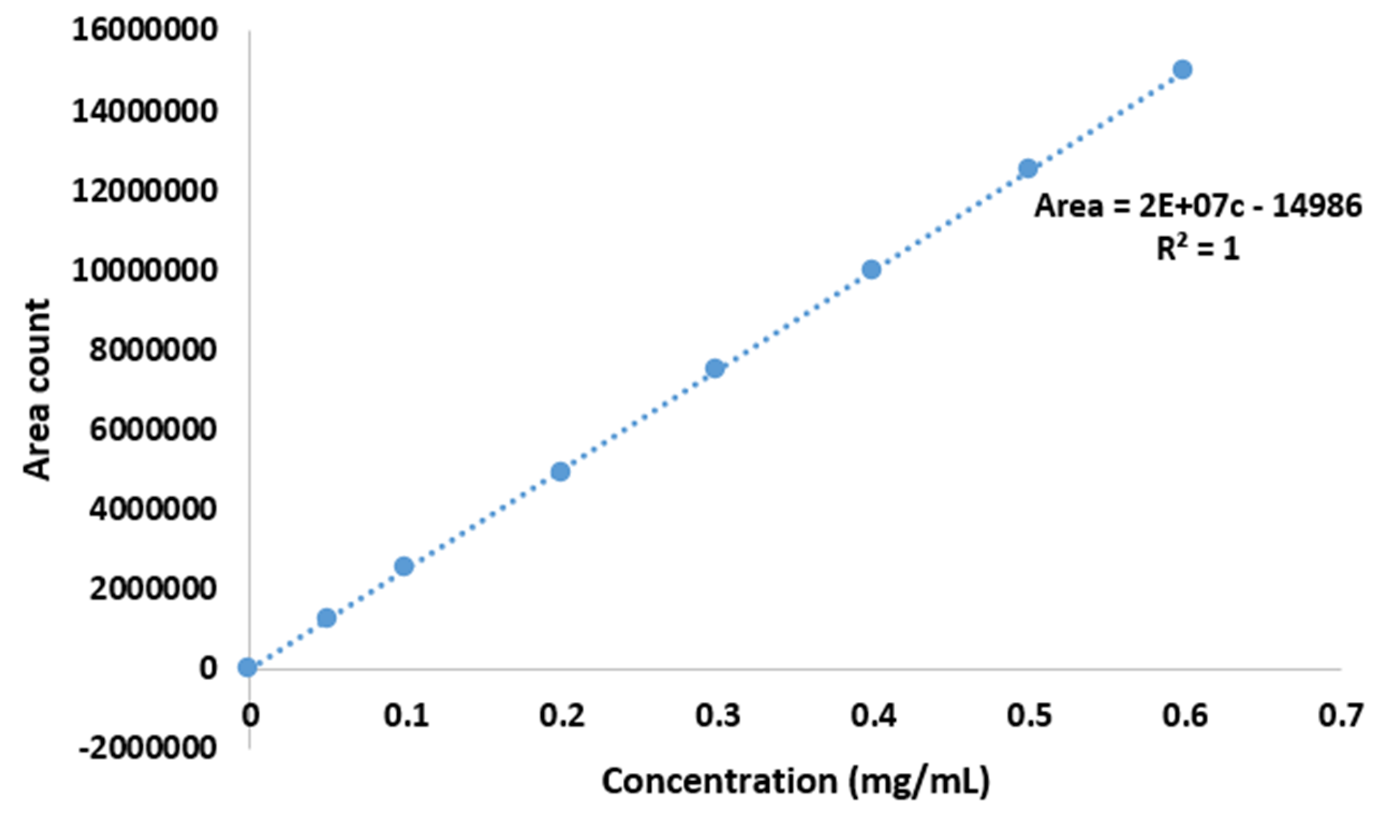

Supplement: S2 Fig — (DOCX) [file pone.0267302.s002.docx]

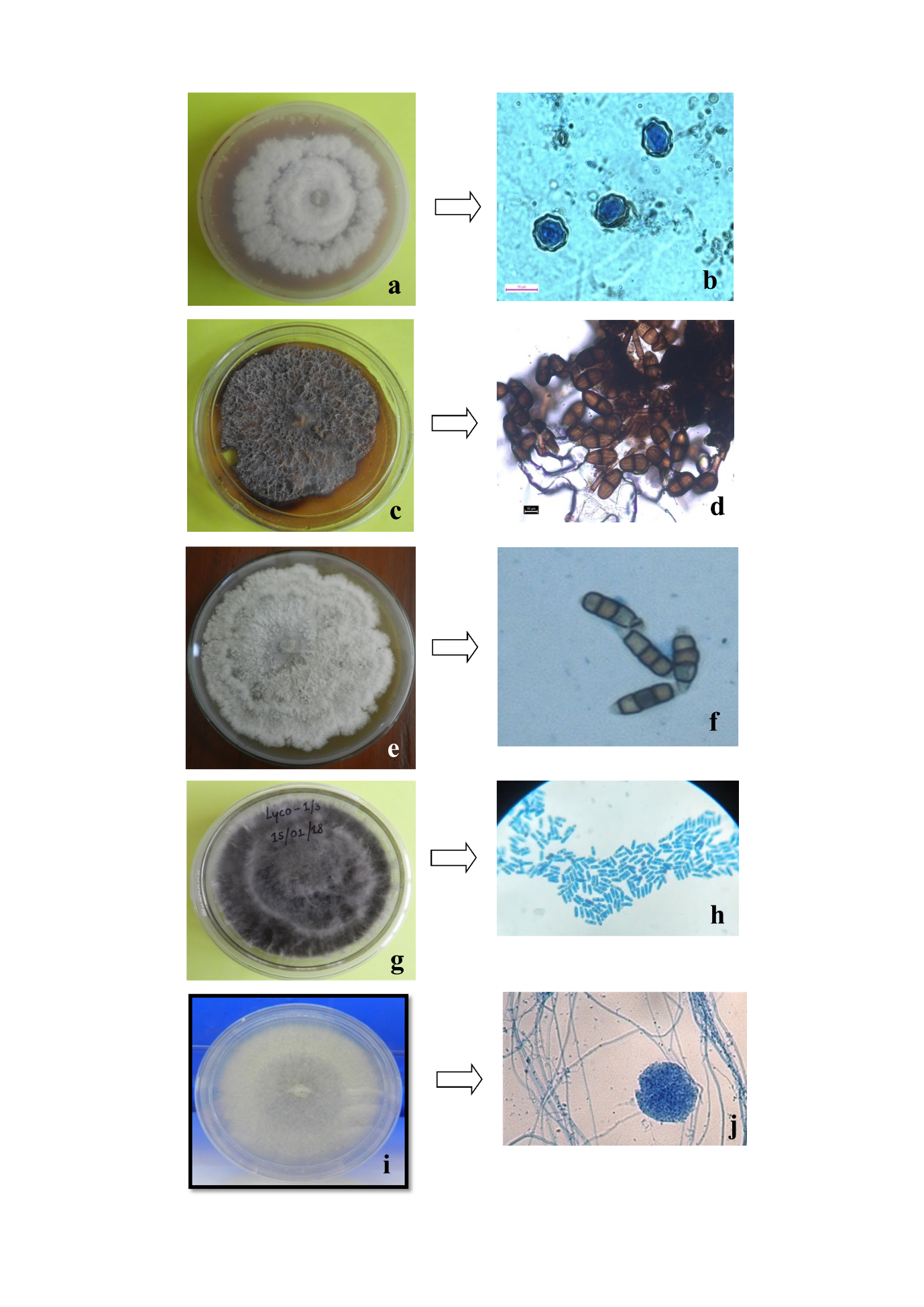

Supplement: S3 Fig — (DOCX) [file pone.0267302.s003.docx]

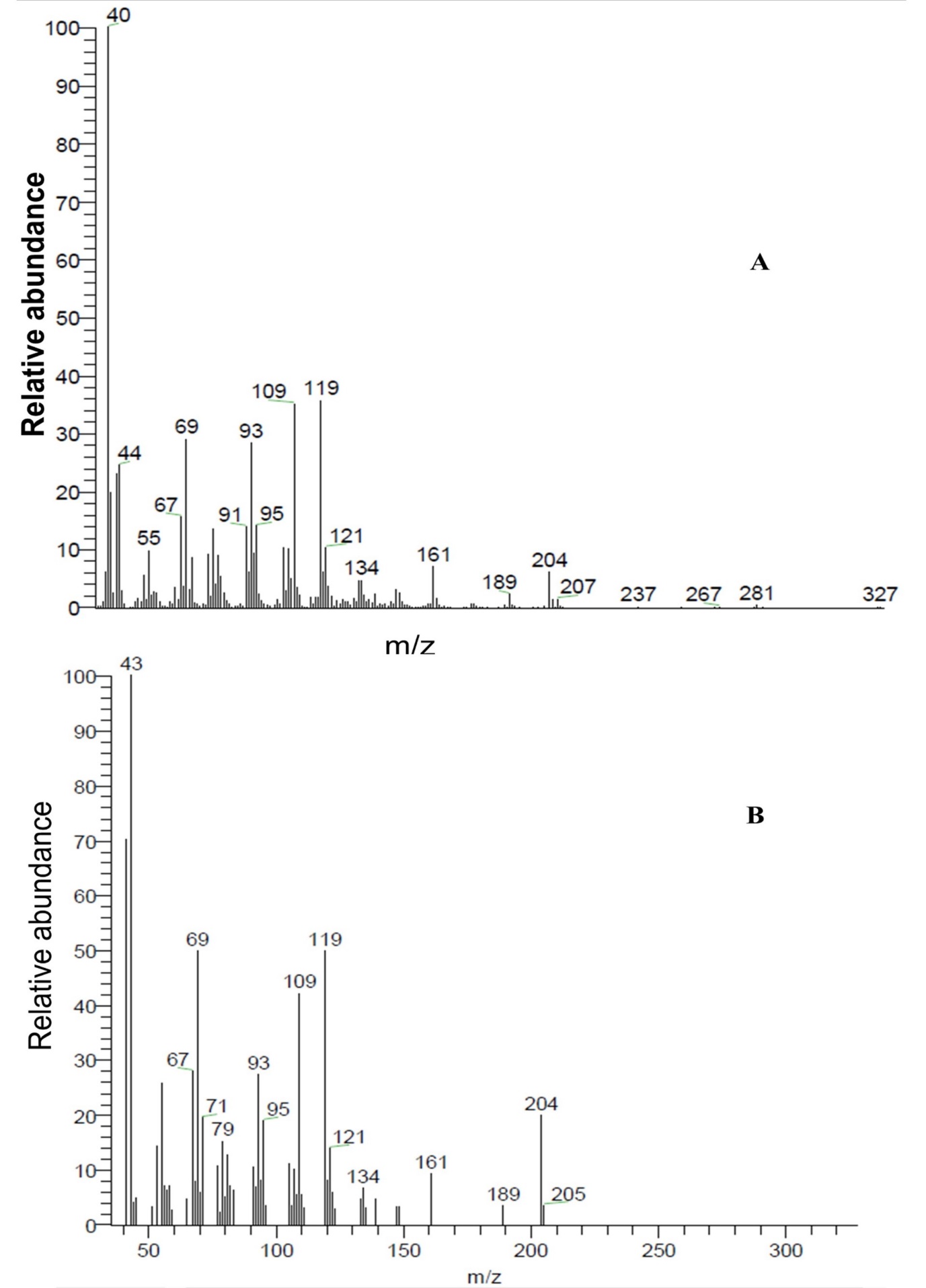

Supplement: S4 Fig — a Electron-ionisation mass spectrum of bisabolol obtained from LCS1 extract. b Electron-ionisation mass spectrum of standard bisabolol. (DOCX) [file pone.0267302.s004.docx]
