# Supplementary material for: Bioactivity study and metabolic profiling of Colletotrichum alatae LCS1, an endophyte of club moss Lycopodium clavatum L
Source: PLoS One. 2022 Apr 28;17(4):e0267302. doi: 10.1371/journal.pone.0267302 (PMC9049576; doi:10.1371/journal.pone.0267302)
Supplement: S1 Table — a. Antibacterial activity (in terms of clear zone of inhibition in mm) of the endophytic isolates against pathogenic bacteria. b MIC values (μg/mL) of culture extract and mycelial extract of Colletotrichum alatae LCS1 obtained using two different types of solvent. (DOCX) [file pone.0267302.s005.docx]

**S1a Table Antibacterial activity (in terms of clear zone of inhibition in mm) of the endophytic isolates against pathogenic bacteria**

| Pathogenic bacteria | *Colletotrichum* sp. | | *Phomopsis* sp. | | *Lasidiplodia* sp. | | *Phoma*  sp. | *Pestaloti opsis* sp. | *Scopulari opsis* sp. | STRP  (10  µg/mL) | CLND  (10  µg/mL) | VANCO  (10  µg/mL) | CPFX  (10  µg/mL) |
| --- | --- | --- | --- | --- | --- | --- | --- | --- | --- | --- | --- | --- | --- |
|  | Withou t P.E. | With P.E. | Without P.E. | With P.E. | Without P.E. | With P.E. |  |  |  |  |  |  |  |
| *B. cereus* (ATCC  14579) | 12.66±  0.58 (a) | 33 (b) | 14.66±0.  58 (a) | 20.33±  0.58 (b) | 14 (a) | 19 (b) | 9.66±0.5  8 (a) | 7.66±0.5  8 (b) | 8 (b) | 21 | 22±0.5  8 | 27 | 24 |
| *B*. *subtilis* (ATCC  11774) | 12.33±  0.58 (a) | 35 (b) | 12.33±0.  58 (a) | 21.66±  0.58 (b) | 14.66±0.  58 (a) | 23 (b) | 10.33±0.  58 (a) | 8.66±0.5  8 (b) | 9.66±0.5  8 (a) | 21 | 21 | 25 | 24 |
| MRSA (ATCC  33591) | 14 (a) | 30 (b) | 12.66±0.  58 (a) | 27 (b) | 11.66±0.  58 (a) | 20 (b) | 10.66±0.  58 (a) | 8.66±0.5  8 (b) | 8.33±058  (b) | 19 | 24±0.5  8 | 23 | 21 |
| *P*. *mirabilis*  (ATCC 12453) | 13.66±  0.58 (a) | 24 (b) | 11.66±0.  58 (a) | 24 (b) | 12.33±0.  58 (a) | 24 (b) | 10 (a) | 12±0.58 (b) | 9 (c) | 18 | 24 | 24 | 21±0.5  8 |
| *P*. *aeruginosa*  (ATCC 9027) | 12 (a) | 30 (b) | 9.66±0.5  8 (a) | 31 (b) | 10.66±0.  58 (a) | 23.66±  0.58 (b) | 11.66±0.  58 (a) | 10.66±0.  58 (b) | 10 (b) | 17 | 29 | 31 | 26 |
| *V*.  *parahaemolyticus*  (ATCC 17802) | 10.33±  0.58 (a) | 23.33±  0.58 (b) | 9.66±0.5  8 (a) | 22.33±  0.58 (b) | 11.66±0.  58 (a) | 27 (b) | 9 (a) | 7.66±0.5  8 (b) | 9 (a) | 16±0.5  8 | 28 | 30±0.58 | 26 |
| *E*. *coli* (MTCC  4296) | 12 (a) | 20 (b) | 11 (a) | 26.66±  0.58 (b) | 10.33±0.58 (a) | 28.33±  0.58 (b) | 10.33±0.  58 (a) | 7.66±0.5  8 (b) | 6.66±0.5  8 (c) | 14 | 29 | 31 | 25 |

For each bacterial pathogen and for each endophytic fungal isolate the two parameters are there (with P.E.-plant extract and with-out P.E.).

The Tukey’s multiple comparison test was done between these two parameters (with P.E. and without P.E.) for each case. There were valid

statistical differences among the data sets (P<0.05), the two different letters a, b indicates significance differences. For the rest of the three isolates

(*Phoma* sp., *Pestalotiopsis* sp. and *Scopulariopsis* sp.) the three different letters a, b, c indicates valid statistical differences and same letter indicates

no statistical differences.

**S1b Table MIC values (µg/mL) of culture extract and mycelial extract of *Colletotrichum alatae* LCS1 obtained using two different types of solvent**

| Pathogenic bacteria | MIC | | | |
| --- | --- | --- | --- | --- |
|  | Ethyl acetate | | Ethyl ether | |
|  | Culture broth | Mycelial extract | Culture broth | Mycelial extract |
| *B. cereus* (ATCC 14579) | 15.62 (a, a_1_) | 125 (b, b_1_) | 125 (c, a_2_) | 250 (d, b_2_) |
| *B*. *subtilis* (ATCC 11774) | 15.62 (a, a_1_) | 125 (b, b_1_) | 125 (c, a_2_) | 250 (d, b_2_) |
| MRSA (ATCC 33591) | 31.25 (a, a_1_) | 250 (b, b_1_) | 250 (c, a_2_) | 500 (d, b_2_) |
| *P*. *mirabilis* (ATCC 12453) | 31.25 (a, a_1_) | 250 (b, b_1_) | 250 (c, a_2_) | 500 (d, b_2_) |
| *P*. *aeruginosa* (ATCC 9027) | 62.5 (a, a_1_) | 500 (b, b_1_) | 500 (c, a_2_) | 1000 (d, b_2_) |
| *V*. *parahaemolyticus* (ATCC 17802) | 62.5 (a, a_1_) | 250 (b, b_1_) | 250 (c, a_2_) | 500 (d, b_2_) |
| *E*. *coli* (MTCC 4296) | 125 (a, a_1_) | 500 (b, b_1_) | 500 (c, a_2_) | 1000 (d, b_2_) |

One way ANOVA (Tukey’s Multiple Comparison test) was performed to check the statistical differences (at P<0.05 level). The letters a, b (in case of ethyl acetate as the extracting agent) and c, d (in case of ethyl ether as the extracting agent) indicates significant statistical differences between MIC values of culture broth and mycelial extracts. The other letters a_1_, a_2_ and b_1_, b_2_ indicates potential statistical differences between MIC of culture broth and mycelial extracts respectively in case of Ethyl acetate and ethyl ether. One way ANOVA (Tukey’s Multiple Comparison test) was performed to check the statistical differences (at P<0.05 level).
